# Electrochemical Performance of TiNb_2_O_7_ Nanofibers for Lithium-Ion Battery Anodes Using Flame-Retardant Electrolytes

**DOI:** 10.3390/ma19091840

**Published:** 2026-04-29

**Authors:** Seongwon Go, Hong Chen, Seul Lee, Garim Lee, Hye Seon Yoon, Minseung Kang, Chae-Ryong Cho

**Affiliations:** 1 Department of Nano Fusion Technology, Pusan National University, Busan 46241, Republic of Korea; gomoti5711@naver.com (S.G.); chenhong4819@163.com (H.C.); seulthink@pusan.ac.kr (S.L.); leegrm@pusan.ac.kr (G.L.); yoonhyeseon0723@naver.com (H.S.Y.); twomin0000@naver.com (M.K.); 2 School of Transdisciplinary Engineering, Pusan National University, Busan 46241, Republic of Korea; 3 Department of Nanoenergy Engineering, Pusan National University, Busan 46241, Republic of Korea

**Keywords:** LIBs, TNO nanofibers, voltage window, electrode-electrolyte interface, flame-retardant electrolyte, thermal safety

## Abstract

This study demonstrates an electrode–electrolyte co-design strategy to address the long-standing performance–safety trade-off in lithium-ion batteries by integrating electrospun TiNb_2_O_7_ (TNO) nanofiber anodes with fluorinated flame-retardant electrolytes. The electrochemical compatibility of TNO was systematically evaluated over a wide voltage window (0.01–3.0 V) using a conventional carbonate electrolyte and two fluorinated systems (TFMAF and NOMAF). At low current densities, the fluorinated electrolytes deliver capacities comparable to those of the carbonate electrolyte, whereas the carbonate system exhibits superior rate capability at high current densities. Among the flame-retardant electrolytes, TFMAF shows slightly improved electrochemical performance, particularly in terms of rate capability and cycling stability. Elevated temperatures enhance ionic conductivity and reduce polarization across all systems, while low-temperature EIS/DRT analysis reveals distinct, electrolyte-dependent differences in interfacial resistance and charge-transfer behavior. Accelerating rate calorimetry confirms that the fluorinated electrolytes significantly improve thermal safety. Notably, NOMAF exhibits superior thermal stability and emerges as a more practical electrolyte candidate due to its enhanced safety and lower cost.

## 1. Introduction

In recent years, fires and explosions caused by thermal runaway in lithium-ion batteries (LIBs) have intensified public safety concerns and underscored the urgent need for battery systems that deliver both high power capability and improved intrinsic safety [1,2,3,4,5,6,7,8,9]. Graphite remains the dominant commercial anode due to its low operating potential (~0.1 V vs. Li/Li^+^), excellent reversibility, and stable cycling performance. However, its limited theoretical capacity (372 mAh g^−1^) and the risk of lithium plating during fast charging remain critical challenges. Lithium deposition under high-rate operation can induce internal short circuits and accelerate thermal runaway, thereby limiting the deployment of LIBs in high-power and fast-charging applications [10,11,12,13].

In this context, materials design strategies have been extensively explored to address these challenges. Beyond the specific context of lithium-ion batteries (LIBs), nanostructured materials have become increasingly important in modern technologies, as their reduced characteristic dimensions, high surface-to-volume ratio, tunable interfaces, and enhanced strain-accommodation capability enable simultaneous regulation of transport kinetics, reaction activity, and structural stability [14]. Early foundational studies established nanoscale engineering as an effective strategy for rechargeable batteries and electrochemical materials, and subsequent developments extended this concept to electrospun nanofiber systems, solar energy conversion, photocatalysis, and water-treatment technologies [15,16,17]. More recent studies and reviews highlight that nanostructured frameworks remain highly relevant for advanced energy-storage devices, particularly where continuous electron/ion pathways, short diffusion lengths, and precise interfacial control are required [18,19].

At the same time, nanostructured semiconductors [20], carbon-based materials, chalcogenides [21], and biogenic nanoparticles [22] are actively being explored for environmental applications, such as the photocatalytic degradation of organic pollutants and water purification, owing to their abundant active sites, tailorable surface chemistry, and enhanced charge-separation behavior. These advances indicate that nanostructure engineering is now recognized as a general materials design principle spanning both energy and environmental technologies, rather than a strategy limited to a single device platform. Among various nanoarchitectures, one-dimensional nanofibers are particularly attractive for battery electrodes because they provide continuous electron-conduction pathways, shortened Li^+^ diffusion distances, and improved electrolyte accessibility.

Among various nanostructured electrode materials, TiNb_2_O_7_ (TNO) has emerged as a promising anode candidate in LIBs owing to its high theoretical capacity (~387 mAh g^−1^), robust structural stability, and relatively high operating potential (~1.6 V vs. Li/Li^+^), which significantly mitigates lithium plating [23,24,25,26]. Furthermore, nanostructuring approaches such as electrospun nanofibers (NFs) can shorten lithium-ion diffusion pathways, enhance electron transport, and improve mechanical integrity, rendering TNO NFs attractive for fast charge–discharge applications [27,28,29,30,31]. Although TNO typically operates above 1.0 V vs. Li/Li^+^, evaluating its electrochemical behavior over an extended voltage window (0.01–3.0 V) enables a systematic assessment of electrolyte reductive stability and interfacial compatibility under low-potential conditions relevant to composite or hybrid anode configurations.

Battery safety is governed not only by electrode materials but also, critically, by electrolyte properties. Conventional carbonate-based electrolytes, while widely adopted in commercial LIBs, exhibit low flash and boiling points and can generate flammable gases upon thermal decomposition, increasing the risk of fire and explosion. Consequently, flame-retardant and non-flammable electrolyte systems have been extensively investigated [32,33,34]. However, many such systems suffer from reduced ionic conductivity and unstable electrode–electrolyte interfacial chemistry, resulting in an inherent performance–safety trade-off. Moreover, because LIBs operate under diverse environmental conditions, maintaining electrochemical performance and safety at elevated temperatures is essential for practical applications [35,36,37,38,39,40,41,42,43,44,45,46,47]. Despite considerable progress, systematic investigations that integrate high-potential anodes with flame-retardant electrolytes under high-temperature operation remain limited [48].

In this study, electrospun TNO nanofibers were employed as the anode active material, and fluorinated flame-retardant electrolytes were introduced to mitigate the performance–safety trade-off across a wide operating voltage window (0.01–3.0 V). Specifically, a commercial carbonate electrolyte (1 M LiPF_6_ in ethylene carbonate (EC)/diethyl carbonate (DEC) = 1:1, *v*/*v*, with 10 wt% fluoroethylene carbonate (FEC)) was compared with fluorinated flame-retardant electrolytes based on 1 M lithium bis(trifluoromethanesulfonyl)imide (LiTFSI) in Bis(2,2,2-trifluoroethyl) carbonate (TFEC)/2,2,2-trifluoro-N,N-dimethylacetamide (FDMA) and methyl nonafluorobutyl ether (NOVEC7200)/FDMA (1:1, *v*/*v*, each containing 10 wt% FEC), hereafter denoted as TFMAF and NOMAF, respectively.

Electrochemical performance was systematically evaluated over a broad temperature range. High-temperature behavior (30–70 °C) was examined to assess rate capability and thermal durability, whereas low-temperature characteristics (−20 to 20 °C) were investigated using electrochemical impedance spectroscopy (EIS). Nyquist plots were analyzed to quantify the evolution of ohmic resistance (R_s_) and charge-transfer resistance (R_ct_) with temperature. Furthermore, distribution of relaxation times (DRT) analysis was employed to deconvolute overlapping impedance contributions and to resolve distinct electrochemical processes. The DRT spectra enabled clear separation of (i) high-frequency features associated with SEI and interfacial film resistance, (ii) mid-frequency peaks corresponding to charge-transfer kinetics at the electrode–electrolyte interface, and (iii) low-frequency components related to diffusion-controlled processes, including Warburg-type lithium-ion transport. This approach provides mechanistic insight into temperature-dependent interfacial kinetics and mass-transport behavior across different electrolyte systems.

Thermal safety was assessed using accelerating rate calorimetry (ARC). By integrating a high-potential, structurally stable TNO nanofiber anode with fluorinated flame-retardant electrolyte chemistries, this work provides practical insights into electrode–electrolyte co-design strategies for next-generation lithium-ion batteries aimed at simultaneously achieving high-power performance, wide-temperature operability, thermal stability, and enhanced intrinsic safety.

## 2. Materials and Methods

### 2.1. Preparation of Electrospun TNO Nanofibers

The precursor solution was prepared by dissolving 0.7 g poly(vinyl pyrrolidone) (PVP) in 9 mL of ethyl alcohol under magnetic stirring for 10 min, followed by the addition of 2 mL of acetic acid and further stirring for 15 min to ensure complete homogenization. The solution was then transferred to an Ar-filled glove box, where titanium(IV) butoxide (0.6 g, Aldrich, St. Louis, MO, USA, reagent grade, 97%) and niobium(V) ethoxide (1.1 g, Thermo Scientific, Waltham, MA, USA, 99.99%) were added to achieve a Ti:Nb:O molar ratio of 1:2:7. The final mixture was stirred at 25 °C for 5 h and used as the electrospinning feed solution. Electrospinning was performed using a syringe equipped with a 25-gauge stainless steel needle. The applied voltage was set to 15 kV, with a controlled flow rate of 2.0 mL h^−1^. A rotating drum collector was operated at 200 rpm, and the tip-to-collector distance was maintained at 20 cm. The as-spun nanofibers were collected on the drum surface, placed in a ceramic crucible, and calcined at 850 °C with a heating rate of 3 °C min^−1^, followed by holding for 5 h to obtain crystalline TNO nanofibers (TNO NFs). The annealing time of 5 h was determined to be the minimum duration required to achieve well-crystallized TNO nanofibers with stable morphology and reproducible electrochemical properties [49,50]. The polymer matrix acted as a structure-directing agent during electrospinning and was completely removed during calcination, resulting in phase-pure oxide nanofibers. The selected calcination temperature and dwelling time were optimized to promote crystallization while preserving the fibrous morphology.

### 2.2. Fabrication of TNO NF Anodes

The anode slurry was prepared by mixing TNO nanofibers, carbon black (MTI Korea, Seoul, Republic of Korea), and a carboxymethyl cellulose (MTI Korea, Seoul, Republic of Korea)/styrene-butadiene rubber(MTI Korea, Seoul, Republic of Korea) (CMC/SBR) binder in a weight ratio of 70:15:15. The active material loading was controlled to ensure reproducible electrochemical performance. The CMC/SBR binder system was selected to provide mechanical flexibility and strong adhesion between the nanofibers and the current collector. The slurry was uniformly coated onto Cu foil using a doctor-blade method. The coated electrodes were initially dried at 70 °C for 6 h in a convection oven, followed by secondary vacuum drying at 60 °C for more than 12 h to remove residual moisture and solvent. The dried electrodes were punched into circular disks with a diameter of 14 mm for electrochemical evaluation. The crystalline structure of the prepared samples was characterized by X-ray diffraction (XRD; D8 VENTURE, Bruker) employing Cu K_α_ radiation (λ = 1.5406 Å). Diffraction patterns were recorded using an X’Pert3 diffractometer (Malvern Panalytical, Almelo, The Netherlands) over a 2θ range of 5–70° at room temperature. Phase identification was performed using X’Pert HighScore 4.9a software based on standard JCPDS/PDF cards. For the comparative analysis between powder and nanofiber samples, a scan step size of 0.013° was applied to ensure high angular resolution. In contrast, a scan step size of 0.026° was adopted for the PVP-related comparison. The surface morphology of the samples was investigated using field-emission scanning electron microscopy (FE-SEM; Supra 40VP, ZEISS) operated at an accelerating voltage of 10 kV. Prior to imaging, the samples were sputter-coated with a thin platinum layer to improve electrical conductivity.

### 2.3. Electrolytes

A commercial carbonate-based reference electrolyte was custom-ordered from Wellcos, consisting of 1 M LiPF_6_ dissolved in (EC/DEC = 1:1, *v*/*v*) with 10 wt% FEC. For the flame-retardant electrolyte systems, LiTFSI was employed as the common lithium salt. Fluorinated solvents, including TFEC, NOVEC7200, and FDMA, were used as flame-retardant components. FEC was added as a common additive for all flame-retardant electrolytes. All flame-retardant electrolytes were synthesized in-house under anhydrous conditions. The use of LiTFSI was intended to enhance thermal and electrochemical stability compared with conventional LiPF_6_-based systems. The electrolyte formulations were designed to balance ionic conductivity with improved flame-retardant characteristics.

Self-extinguishing time (SET) tests were conducted to evaluate the flame-retardant properties of the electrolytes. A 50 μL aliquot of electrolyte was dropped onto a stainless-steel cell can. A glass fiber separator was immersed in the electrolyte for 30 s to ensure complete wetting, then fixed vertically using a clamp stand and exposed to an external flame generated by a torch, with a fixed distance of 1 cm between the torch nozzle and the separator. The SET was defined as the time interval between ignition of the separator and complete flame extinction, with no visible combustion remaining.

The room-temperature ionic conductivities of EDF, NOMAF, and TFMAF were measured by electrochemical impedance spectroscopy (EIS) using stainless-steel (SS)|stainless-steel (SS) blocking cell. The measurements were performed with an amplitude of 10 mV over a frequency range from 1 MHz to 0.01 Hz. The ionic conductivity was calculated using the following Equation (1):

(1) $$\sigma =\frac{d}{R\times A}$$

where d is the thickness of the separator, R is the bulk electrolyte resistance obtained from the Nyquist plot, and A is the effective area of the separator.

The Li^+^ transference number ($t_{Li^{+}}$) was determined using a combination of a potentiostatic polarization method and EIS measurements with symmetric Li|electrolyte|Li cells. A constant direct current polarization of 10 mV was applied, and the initial current ($I_{0}$) and steady-state current ($I_{s}$) were recorded. The interfacial resistances before and after polarization were obtained from EIS measurements as $R_{0}$ and $R_{s}$, respectively. The Li^+^ transference number was calculated according to Equation (2) [51]:

(2) $$t_{Li^{+}}=\frac{I_{s}(\Delta V-I_{0}R_{0})}{I_{0}(\Delta V-I_{s}R_{s})}$$

where ΔV  is the applied direct current polarization voltage, $I_{0}$ and $I_{s}$ are the initial and steady-state currents, respectively, and $R_{0}$ and $R_{s}$ are the interfacial resistances measured before and after polarization.

Accelerating rate calorimetry (ARC) measurements were performed using a BTC-130 calorimeter (H.E.L Ltd., Hemel Hempstead, UK). The ARC experiments were conducted in a spherical quarter-cell test bomb supplied by the manufacturer. The electrolyte sample volume for ARC testing was 2 mL. The initial temperature was set to 40 °C. Experiments were carried out in heat–wait–search (HWS) mode with a temperature increment of 10 °C per step, a waiting (equilibration) time of 30 min, and a search time of 5 min. The maximum temperature and pressure limits were set to 350 °C and 150 bar, respectively. Self-heating was defined as a temperature rise rate exceeding 0.03 °C min^−1^.

### 2.4. Cell Assembly and Electrochemical Measurements

Electrochemical performance was evaluated using CR2032 coin-type half-cells assembled in an Ar-filled glove box (O_2_ and H_2_O levels < 0.1 ppm). Lithium metal foil (thickness: 700 μm, diameter: 16 mm) served as the counter and reference electrode. A trilayer polypropylene–polyethylene–polypropylene (PP-PE–PP) separator with a diameter of 19 mm was used. After assembly, the cells were allowed to rest for 15 h prior to testing to ensure interfacial equilibration and stabilization between the electrodes and electrolytes. Galvanostatic charge–discharge (GCD), rate capability, long-term cycling, and cyclic voltammetry (CV) measurements were performed using an automatic battery testing system (WBCS3000Le32, WonaTech) at 30 °C. The GCD tests were conducted within voltage windows of 0.01–3.0 V and 1.0–3.0 V. For the rate capability tests, the cells were cycled at current densities of 0.1, 0.5, 1, 3, 5, and 10 A g^−1^ for 10 cycles at each current density, followed by a reverse current sequence. For the evaluation of long-term cycling stability, the cells were initially subjected to three formation cycles at 0.1 A g^−1^ and subsequently cycled at 5 A g^−1^ for 1000 cycles. CV measurements were carried out within voltage windows of 0.01–3.0 V and 1.0–3.0 V at a scan rate of 0.1 mV s^−1^. Additional scan rate-dependent CV tests were performed at 0.1, 0.5, 1, 3, 4, and 5 mV s^−1^. The b-values were calculated from the main anodic and cathodic peaks at lower scan rates. Electrochemical impedance spectroscopy (EIS) measurements were performed using a multi-channel electrochemical workstation (ZIVE MP1, WonaTech) with an AC amplitude of 10 mV over a frequency range from 1000 kHz to 0.1 Hz. The raw impedance data, including frequency, real impedance (Z_real_), and imaginary impedance (Z_im_), were extracted and analyzed using EIS analysis software (RelaxIS 3, rhd instruments, Darmstadt, Germany) to obtain the distribution of relaxation times (DRT) spectra. The deconvolution was performed via Tikhonov regularization, where the regularization parameter (λ) was optimized using the L-curve method, and a Gaussian basis function was used for discretization of the timescale. The DRT analysis enabled the deconvolution of overlapping electrochemical processes, such as interfacial resistance and charge-transfer reactions. To assess reproducibility, key electrochemical measurements were repeated using independently assembled cells under identical conditions (*n* ≥ 3), and the corresponding values are reported with associated error ranges (≤±3%).

### 2.5. Electrode Characterization After Cycling

After 1000 cycles, X-ray diffraction (XRD) and X-ray photoelectron spectroscopy (XPS) analyses were performed to investigate the structural stability and surface chemistry of the TNO electrodes following electrochemical cycling. The pristine TNO electrode and cycled TNO electrodes recovered from cells using EDF, NOMAF, and TFMAF electrolytes were compared. After cycling, the cells were disassembled in an Ar-filled glovebox, and the electrodes were gently rinsed with anhydrous dimethyl carbonate (DMC) to remove residual electrolyte, followed by vacuum drying. The dried electrodes were then transferred for characterization with minimal air exposure. XRD measurements were conducted using an X-ray diffractometer (D8 VENTURE, Bruker) with Cu Kα radiation (λ = 1.5406 Å) at room temperature. XPS measurements were carried out using an XPS system equipped with Al K_α_ radiation (hν = 1486.6 eV). Survey spectra and high-resolution spectra of C 1s, O 1s, F 1s, N 1s, Ti 2p, Nb 3d, S 2p, P 2p, and Li 1s were collected. The binding energies were calibrated using the C 1s peak at 284.8 eV as the reference.

## 3. Results and Discussion

**Figure 1:**
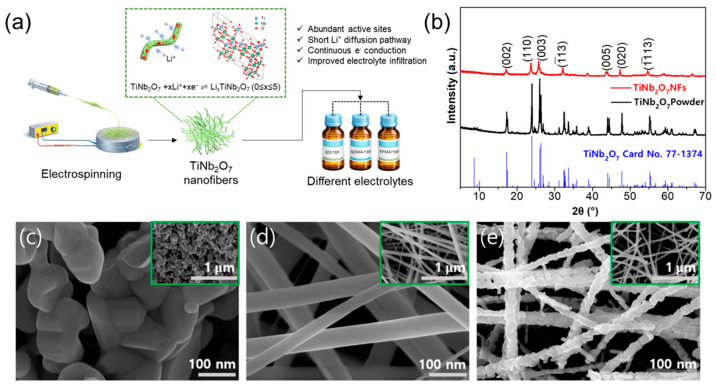
(**a**) Schematic illustration of the sample preparation process. (**b**) X-ray diffraction (XRD) patterns of TiNb_2_O_7_ (TNO) nanofibers (NFs) and TNO powder, together with the standard reference pattern of TNO. Scanning electron microscopy (SEM) images of (**c**) TNO powder, (**d**) TNO NFs before annealing, and (**e**) TNO NFs after annealing.

TNO NFs were prepared via an electrospinning-assisted route followed by thermal treatment to remove the polymeric matrix and promote crystallization. Figure 1 provides an overview of the material design concept and the structural/morphological characterization. As schematically illustrated in Figure 1a, the electrospun one-dimensional nanofiber architecture is expected to offer abundant electrochemically active sites, shortened Li^+^ diffusion pathways, continuous electron-conduction networks, and improved electrolyte infiltration, enabling a meaningful comparison of different electrolyte chemistries in this work. The phase formation of the TNO NFs was first verified by XRD. Figure 1b compares the XRD patterns of the TNO nanofibers and TNO powder with the standard reference card. The diffraction peaks in the 2θ range of 5–70° match well with monoclinic TiNb_2_O_7_ (space group C2/m, No. 12; JCPDS card No. 77-1374), consistent with reported lattice parameters (a = 20.351 Å, b = 3.801 Å, c = 11.882 Å, α = 90°, β = 120.19°, and γ = 90°). No discernible impurity peaks were observed, indicating successful formation of phase-pure TNO in the nanofiber form. Compared with the powder, the nanofibers show comparable peak positions, confirming that the electrospinning/annealing process preserves the TNO crystal structure. Figure S1 presents additional XRD patterns obtained under different PVP contents and calcination temperatures. The morphology was examined by SEM. Figure 1c shows the TNO powder composed of agglomerated submicron particles. In contrast, the electrospun sample before annealing exhibits a continuous one-dimensional fibrous network with smooth surfaces and an interconnected structure (Figure 1d). After annealing, the overall fibrous morphology is preserved; however, the fiber surfaces become noticeably rougher (Figure 1e), indicating crystallization and grain growth along the fiber framework. The insets show low-magnification views of the corresponding samples. Overall, the electrospun nanofibers form a uniform and interconnected network structure suitable for subsequent electrochemical evaluation.

**Figure 2:**
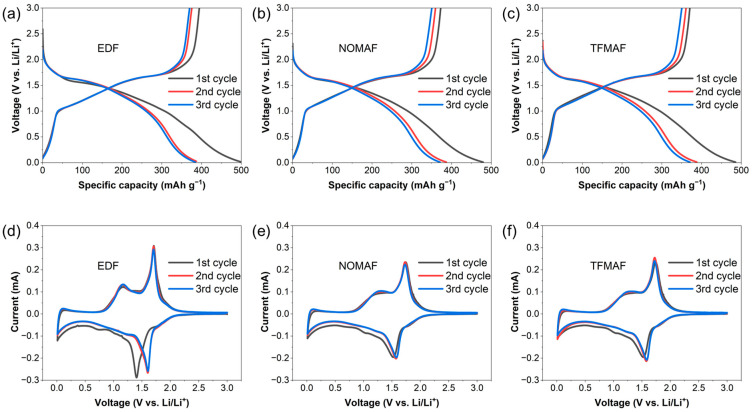
(**a**–**c**) Galvanostatic charge/discharge (GCD) voltage profiles of TNO NF anodes measured in three different electrolytes (EDF, NOMAF, and TFMAF) for the first three cycles at a current density of 0.1 A g^−1^ within a potential window of 0.01–3.0 V vs. Li/Li^+^. (**d**–**f**) Cyclic voltammetry (CV) curves for the first three cycles recorded at a scan rate of 0.1 mV s^−1^ in EDF, NOMAF, and TFMAF, respectively.

Figure 2a–c show the galvanostatic charge/discharge (GCD) profiles of TNO nanofiber (NF) electrodes measured at 0.1 A g^−1^ within the voltage window of 0.01–3.0 V using different electrolytes. All three systems exhibit similar voltage plateaus and overall profile shapes during the first three cycles, indicating that the lithium insertion/extraction mechanism of TNO is maintained irrespective of electrolyte chemistry. The initial discharge capacity of EDF is 501 mAh g^−1^, while NOMAF and TFMAF deliver comparable values of 479 and 485 mAh g^−1^, respectively. The capacity difference between the first and second discharge cycles for both voltage windows is shown in Table S1. A slightly increased polarization between charge and discharge curves is observed for the flame-retardant electrolytes during the first cycle, which may be associated with their relatively lower ionic conductivity and interfacial resistance compared with the conventional carbonate system. However, this polarization difference diminishes in subsequent cycles, suggesting progressive stabilization of the electrode–electrolyte interface. The overlapping second and third cycle profiles further indicate stable electrochemical behavior after initial activation. To elucidate the structural contribution of the nanofiber architecture under restricted lithiation conditions, galvanostatic charge–discharge (GCD) profiles of TNO powder (Figure S2a–c) and TNO nanofiber (NF) electrodes (Figure S2d–f) were compared within a limited voltage window of 1.0–3.0 V. Notably, the capacity reduction observed in flame-retardant electrolytes is more pronounced for powder electrodes than for NFs. This contrast suggests that the interconnected one-dimensional nanofiber framework enhances charge transport and mitigates kinetic constraints, particularly under electrolyte systems with comparatively sluggish transport properties [52,53].

Figure 2d–f present the cyclic voltammetry (CV) curves recorded at 0.1 mV s^−1^ between 0.01 and 3.0 V (vs. Li/Li^+^). All electrolyte systems display comparable redox features, confirming similar electrochemical reaction pathways. For EDF, distinct redox peaks appear at approximately 1.7 V during anodic scanning and 1.4 V during cathodic scanning, corresponding to the Nb^5+^/Nb^4+^ redox transition. The weaker features observed in the 0.6–0.8 V range are associated with the Nb^4+^/Nb^3+^ reaction, while the broad signals above 1.75 V are attributed to overlapping Ti^4+^/Ti^3+^ redox contributions and multi-step lithium insertion processes [54,55]. The cathodic peak in the first cycle differs from those in the subsequent cycles, which can be attributed to initial structural rearrangement of TNO and the formation of a solid electrolyte interphase (SEI). From the second cycle onward, the CV curves nearly overlap, indicating improved reversibility and stable interfacial behavior after the initial activation process. Compared with EDF, NOMAF and TFMAF exhibit smaller variations in peak position between cycles, implying relatively stable interfacial evolution during early cycling despite the use of flame-retardant solvents.

To further elucidate the kinetic characteristics, additional CV measurements were conducted within a restricted voltage window of 1.0–3.0 V at the same scan rate of 0.1 mV s^−1^ (Figure S3). The overall redox features remain consistent with those observed over the full voltage range, confirming that the dominant charge-storage mechanism is preserved. The scan-rate-dependent current response further supports the mixed diffusion–pseudocapacitive behavior discussed below.

Overall, these electrochemical results demonstrate that the TNO nanofiber architecture maintains comparable lithium storage behavior across different electrolyte chemistries while partially compensating for the kinetic limitations associated with flame-retardant electrolyte systems.

**Figure 3:**
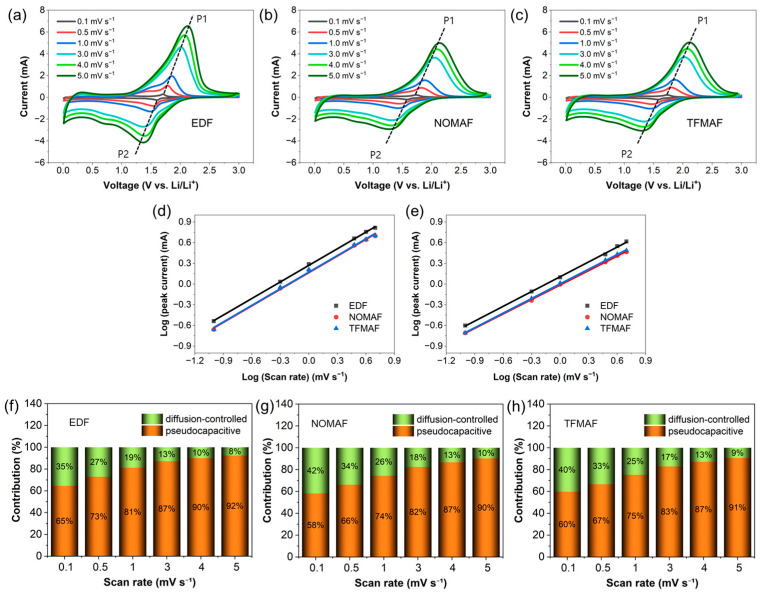
(**a**–**c**) Cyclic voltammetry (CV) curves recorded at scan rates ranging from 0.1 to 5.0 mV s^−1^ for cells employing EDF, NOMAF, and TFMAF electrolytes, respectively, measured within a potential window of 0.01–3.0 V vs. Li/Li^+^. (**d**,**e**) Log–log relationships between peak current and scan rate, together with the corresponding *b*-values for the oxidation (P1) and reduction (P2) peaks, as marked in (**a**–**c**). (**f**–**h**) Quantitative contributions of pseudocapacitive and diffusion-controlled charge-storage processes for EDF, NOMAF, and TFMAF electrolytes at scan rates from 0.1 to 5.0 mV s^−1^, calculated based on Dunn’s method.

Figure 3 presents a systematic scan-rate-dependent kinetic analysis of TNO nanofiber (NF) anodes operated in EDF, NOMAF, and TFMAF electrolytes within a potential window of 0.01–3.0 V vs. Li/Li^+^. Figure 3a–c compare the corresponding cyclic voltammetry (CV) responses recorded at scan rates ranging from 0.1 to 5.0 mV s^−1^. With increasing scan rate, both anodic and cathodic peak currents increase together with peak broadening and gradual potential shifts, reflecting enhanced polarization arising from coupled ion-transport and interfacial charge-transfer limitations under accelerated electrochemical operation. These features indicate increasing kinetic polarization associated with transport limitations, while surface-controlled processes progressively dominate the charge-storage response at higher scan rates. Among the three electrolytes, EDF consistently exhibits higher peak currents, particularly at elevated scan rates, indicating improved interfacial kinetics and enhanced ionic accessibility, which suggests a relatively greater contribution from surface-controlled charge storage under dynamic conditions. Despite lower peak currents, the flame-retardant electrolytes maintain similar kinetic trends, indicating preserved charge-storage mechanisms. To quantitatively probe the reaction kinetics, the relationship between peak current (*i*) and scan rate (*ν*) was analyzed using the power-law equation (*i* = a*νᵇ*), and the corresponding log(*i*)–log(*ν*) plots are presented in Figure 3d,e. The extracted *b*-values are 0.80 (EDF), 0.80 (NOMAF), and 0.80 (TFMAF) for P1, and 0.71 (EDF), 0.70 (NOMAF), and 0.71 (TFMAF) for P2. Since a *b*-value of 0.5 corresponds to diffusion-controlled intercalation, whereas values approaching unity indicate surface-controlled behavior, the observed range (0.70–0.80) confirms a pseudocapacitive charge-storage mechanism [56,57]. Although the b-values are similar, indicating that the fundamental charge-storage mechanism is preserved, electrolyte chemistry modulates the relative contributions and efficiencies of diffusion- and surface-controlled processes.

To further evaluate whether this kinetic interpretation depends on the selected voltage window, additional analyses were conducted within a narrower range of 1.0–3.0 V (Figures S3 and S4). The resulting *b*-values—0.81 (EDF), 0.79 (NOMAF), and 0.77 (TFMAF) for P1, and 0.74 (EDF), 0.73 (NOMAF), and 0.71 (TFMAF) for P2—are consistent with those obtained over the wider 0.01–3.0 V range. This close agreement confirms that the kinetic behavior is essentially independent of the applied voltage window. Furthermore, it indicates that narrowing the voltage range from 0.01 to 3.0 V to 1.0–3.0 V does not significantly alter the underlying charge-storage mechanism. Instead, the dominant kinetic contribution arises primarily from reactions within the 1.0–3.0 V region, while processes in the low-potential region exert only a negligible influence on the overall storage mechanism.

Further quantitative separation of diffusion-controlled and surface-controlled processes was performed using Dunn’s method [58,59,60] (*i*(V) = k_1_*ν* + k_2_*ν*^1/2^), and the results are summarized in Figure 3f–h. In the full voltage window (0.01–3.0 V), the pseudocapacitive contribution increases monotonically with scan rate, rising from 65% to 92% for EDF, 58% to 90% for NOMAF, and 60% to 91% for TFMAF as the scan rate increases from 0.1 to 5 mV s^−1^. A comparison with the restricted voltage window (1.0–3.0 V, Figure S4f–h) further clarifies the influence of low-potential reactions. EDF exhibits nearly identical capacitive contributions (65% → 92%) in both voltage ranges, whereas NOMAF and TFMAF show slightly reduced capacitive fractions at low scan rates (57% and 52% at 0.1 mV s^−1^, respectively), followed by similarly high contributions at fast scan rates (88–87% at 5 mV s^−1^). These results indicate that exclusion of the low-potential region slightly modifies the relative diffusion–capacitive balance under slow polarization conditions, but does not alter the overall scan-rate-dependent kinetic evolution. Representative deconvolution profiles at 3.0 mV s^−1^ (Figure S5) further visualize the spatial distribution of kinetic contributions. Comparable pseudocapacitive regions are preserved in both the full and restricted voltage windows, confirming that surface-controlled reactions contribute substantially to charge storage irrespective of voltage range.

The slightly reduced pseudocapacitive fraction observed for NOMAF and TFMAF at low scan rates upon narrowing the voltage window suggests that reactions occurring below 1.0 V may partially contribute to surface-associated currents in these flame-retardant electrolytes, possibly related to electrolyte-dependent interphase processes. In contrast, EDF exhibits nearly unchanged behavior between the two voltage windows, indicating that its surface-controlled charge storage predominantly originates from reactions within the 1.0–3.0 V region. Importantly, at elevated scan rates all electrolyte systems converge to similarly high pseudocapacitive contributions (~87–92%), demonstrating that the dominant charge-storage mechanism remains governed by robust mixed diffusion–pseudocapacitive kinetics regardless of voltage-window selection.

Collectively, the strong agreement among *b*-value analysis, Dunn quantification, and voltage-window-dependent validation confirms that electrolyte chemistry primarily modulates the relative diffusion–capacitive balance rather than altering the fundamental charge-storage mechanism, with EDF exhibiting a consistently more favorable diffusion–capacitive balance across independent kinetic analyses.

**Figure 4:**
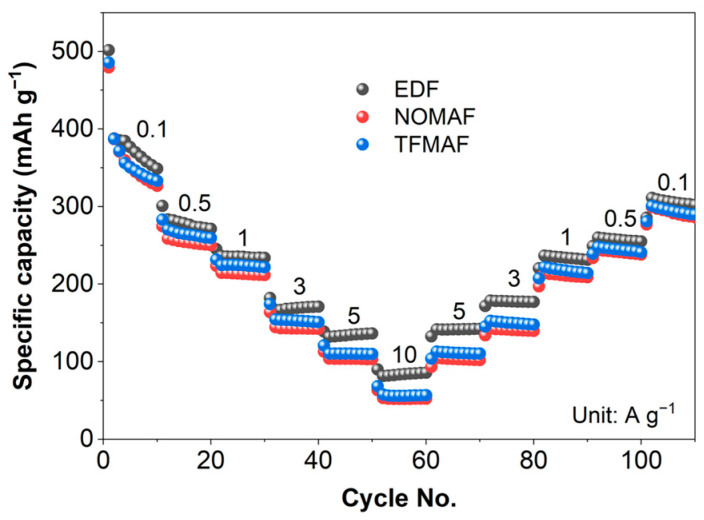
Rate capability of TNO NF anodes in EDF, TFMAF, and NOMAF electrolytes within a voltage window of 0.01–3.0 V (vs. Li/Li^+^) under stepwise current densities ranging from 0.1 to 10 A g^−1^, followed by a return to 0.1 A g^−1^. A progressive decline in specific capacity is observed with increasing current density, while substantial capacity recovery upon returning to 0.1 A g^−1^ demonstrates excellent rate reversibility and structural stability.

The rate capability results shown in Figure 4 reveal electrolyte-dependent kinetic behavior within a voltage window of 0.01–3.0 V vs. Li/Li^+^. As the current density increased from 0.1 to 10 A g^−1^, the discharge capacities gradually decreased for all cells and subsequently recovered upon returning to 0.1 A g^−1^, indicating largely reversible polarization during high-rate cycling. As summarized in Table S2, EDF delivered discharge capacities of 387, 283, 236, 166, 132, and 81 mAh g^−1^ at current densities from 0.1 to 10 A g^−1^. Under identical conditions, NOMAF and TFMAF exhibit moderately lower capacities than EDF, with TFMAF consistently delivering slightly higher capacities than NOMAF while maintaining similar rate-dependent behavior. Even at 10 A g^−1^, capacities of 53–57 mAh g^−1^ were retained, corresponding to approximately 65–70% of the carbonate-based EDF system, demonstrating that the rate capability remains largely preserved despite incorporation of flame-retardant components. Upon restoring the current density to 0.1 A g^−1^, the discharge capacities recovered to 311, 299, and 300 mAh g^−1^ for EDF, NOMAF, and TFMAF, corresponding to capacity retentions of 80.3%, 77.4%, and 77.5%, respectively. The comparable recovery behavior confirms the absence of severe irreversible degradation during high-rate cycling and indicates stable electrode–electrolyte interfacial evolution. Consistent with the kinetic analyses in Figure 3, the superior rate performance of EDF correlates with its higher *b*-values and larger pseudocapacitive contributions, reflecting more efficient surface-controlled charge storage. Importantly, the preserved high-rate capability in NOMAF and TFMAF further demonstrates that mixed diffusion–pseudocapacitive kinetics effectively mitigate the kinetic penalties typically associated with flame-retardant electrolyte systems.

To further clarify the structural origin of rate performance, Figure S6 compares the rate capability of TNO nanofiber (NF) and powder anodes in EDF, NOMAF, and TFMAF electrolytes under stepwise current densities ranging from 0.1 to 10 A g^−1^, followed by recovery at 0.1 A g^−1^, evaluated within voltage windows of 0.01–3.0 V and 1.0–3.0 V vs. Li/Li^+^. As expected, capacities measured within the restricted 1.0–3.0 V window are lower due to exclusion of the low-potential lithiation region. Under this condition, EDF retains 67 mAh g^−1^ at 10 A g^−1^, whereas NOMAF and TFMAF deliver 13 and 14 mAh g^−1^, respectively, while maintaining comparable low-rate capacities (~277–286 mAh g^−1^) with high retention values of 90.6–98.1%. Across all electrolyte systems, NF electrodes consistently exhibit higher capacities than powder electrodes over the entire current-density range, with the performance advantage becoming increasingly pronounced in flame-retardant electrolytes at high rates. This behavior indicates that the continuous one-dimensional NF architecture effectively alleviates transport limitations associated with reduced ionic conductivity and interfacial kinetics. The interconnected NF network shortens ion/electron transport pathways and enables efficient utilization of fast surface-controlled charge-storage processes identified in the kinetic analysis, thereby mitigating rate limitations under demanding operating conditions.

Overall, although flame-retardant electrolytes exhibit moderately lower specific capacities than the conventional carbonate-based system, the kinetic penalty remains limited under practical operating conditions. The maintained reversible capacities and high retention behavior demonstrate that flame-retardant electrolyte systems can preserve viable rate performance while simultaneously offering enhanced safety characteristics.

**Figure 5:**
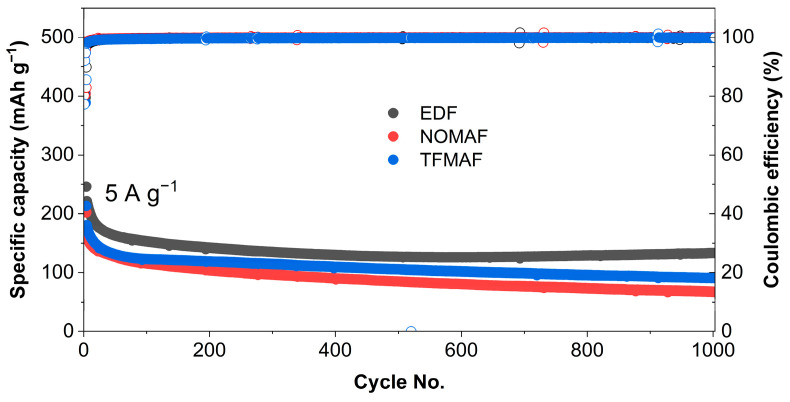
Long-term cycling performance and corresponding Coulombic efficiency of TNO NF anodes in EDF, NOMAF, and TFMAF electrolytes over 1000 cycles at a current density of 5 A g^−1^.

Long-term cycling at a high current density (Figure 5) more clearly highlights electrolyte-dependent electrochemical stability under kinetically demanding conditions. EDF maintains the highest reversible capacity over 1000 cycles, delivering approximately 246 mAh g^−1^ at the initial stage and retaining 133 mAh g^−1^ after 1000 cycles, corresponding to a capacity retention of 54%. In comparison, NOMAF exhibits a lower yet relatively stable capacity, decreasing from 202 to 67 mAh g^−1^ after 1000 cycles (33% retention), while TFMAF declines from 213 to 90 mAh g^−1^ over the same period (42% retention). The Coulombic efficiency (CE) of all three electrolytes rapidly stabilizes near 100% after the initial cycles, indicating highly reversible Li^+^ storage with stabilized electrode–electrolyte interfacial reactions during prolonged high-rate operation. Despite the lower retained capacities observed in the fluorinated flame-retardant electrolytes, their stable cycling behavior demonstrates that long-term electrochemical reversibility is largely preserved. Overall, Figure 5 shows that the carbonate-based EDF electrolyte provides superior capacity retention under aggressive cycling conditions, whereas TFMAF demonstrates better long-term cycling stability than NOMAF among the fluorinated flame-retardant electrolytes while still offering intrinsic safety advantages. These results highlight the performance–safety trade-off governed by electrolyte chemistry and establish a basis for optimizing balanced electrochemical performance and thermal safety. To further examine the structural origin of long-term cycling stability, Figure S7 compares the cycling performance of TNO nanofiber (NF) and powder anodes over 1000 cycles at a current density of 5 A g^−1^ in EDF, NOMAF, and TFMAF electrolytes under both 0.01–3.0 V and 1.0–3.0 V voltage windows. As expected, the initial capacities measured within the restricted 1.0–3.0 V window are significantly lower than those obtained over 0.01–3.0 V due to exclusion of the low-potential lithiation region. Under this limited voltage range, EDF delivers an initial capacity of approximately 210 mAh g^−1^ and gradually decreases to 100 mAh g^−1^ after 1000 cycles, whereas NOMAF and TFMAF start at approximately 90–100 mAh g^−1^ and decay to 30–40 mAh g^−1^ over the same period. Despite the reduced absolute capacities, the relative stability trend (EDF > TFMAF ≥ NOMAF) remains unchanged. The detailed discharge capacities at the 1st and 1000th cycles, together with the corresponding capacity retention values under both voltage windows, are summarized in Table S3.

Across all electrolyte systems and voltage ranges, NF electrodes exhibit higher reversible capacities and improved capacity retention compared with powder electrodes. The performance gap becomes increasingly pronounced in flame-retardant electrolytes (NOMAF and TFMAF), where powder electrodes undergo rapid initial capacity decay and continuous degradation during extended high-rate cycling. In contrast, NF electrodes maintain more stable cycling behavior, indicating enhanced structural robustness and interfacial stability under kinetically demanding conditions. Even within the restricted 1.0–3.0 V window, where absolute capacities decrease due to exclusion of the low-potential lithiation region, the superiority of the NF architecture remains evident and the relative electrolyte stability trend is preserved. These results suggest that the continuous one-dimensional nanofiber architecture effectively suppresses structural and interfacial degradation during prolonged high-rate operation, particularly in flame-retardant electrolyte environments. To further support these observations, Figure S8 presents the corresponding SEM images of the electrodes after formation and after 1000 cycles in different electrolytes, enabling a direct comparison of morphological evolution under varying electrolyte and cycling conditions.

To investigate the origin of the electrolyte-dependent cycling behavior shown in Figure 5, XRD and XPS analyses were performed on TNO electrodes after 1000 cycles in EDF, NOMAF, and TFMAF electrolytes. The XRD results (Figure S9) show that the TNO structure remains unchanged during cycling, indicating the absence of bulk degradation. The XPS analysis (Figure S10) reveals the formation of electrolyte-derived species, such as carbonyl- and carbonate-type compounds and Li–O species, suggesting the establishment of a passivating SEI/interphase layer. EDF forms a carbonate-rich interphase, which contributes to favorable interfacial contact and Li^+^ transport, whereas TFMAF forms a LiF-rich interphase, promoting improved cycling stability. The surface chemistry of TNO evolves differently depending on the electrolyte composition, with distinct interphase formations observed for each electrolyte.

To systematically elucidate the origin and evolution of impedance in TNO electrodes operated with different electrolyte systems, electrochemical impedance spectroscopy (EIS) combined with distribution of relaxation times (DRT) analysis was employed. Compared with kinetic information inferred solely from galvanostatic charge–discharge measurements, EIS enables simultaneous evaluation of ohmic resistance, interphase-related processes, interfacial charge transfer, and mass-transport polarization, thereby providing direct insight into the dominant sources of electrochemical polarization [61]. However, in practical electrode systems, multiple electrochemical processes frequently overlap in the frequency domain, rendering mechanistic interpretation based solely on qualitative Nyquist-plot inspection or equivalent-circuit fitting ambiguous due to parameter coupling and non-uniqueness [62]. To overcome these limitations, DRT analysis was introduced as an advanced impedance-analysis approach capable of separating overlapping relaxation phenomena with minimal reliance on predefined circuit assumptions [63]. Conceptually, DRT represents the measured impedance spectrum as a superposition of elementary relaxation processes distributed over characteristic time constants, where each resolved peak corresponds to dominant electrochemical processes such as interphase transport, charge-transfer reactions, or solid-state diffusion. Accordingly, Nyquist plots and DRT spectra are analyzed together in the following section to clarify the distribution of impedance contributions and their cycling-induced evolution in different electrolyte systems at room temperature.

**Figure 6:**
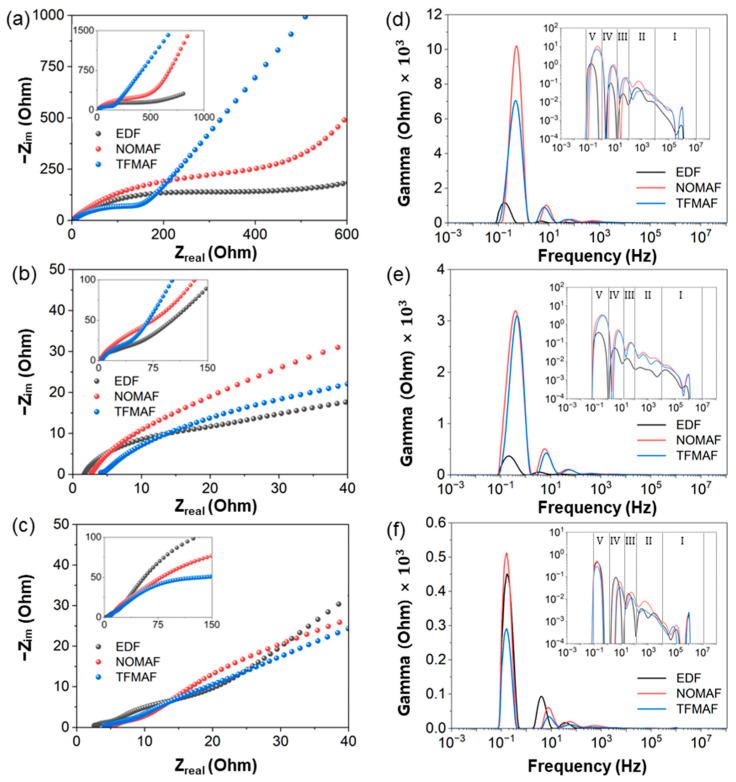
Electrochemical impedance spectroscopy (EIS) Nyquist plots and the corresponding distribution of relaxation time (DRT) analyses of cells with different electrolytes, measured within a voltage window of 0.01–3.0 V. Nyquist plots are shown for (**a**) fresh cells (before cycling), (**b**) after formation, and (**c**) after 1000 cycles at 5 A g^−1^. The insets in (**a**–**c**) present the impedance responses over an extended impedance range. Corresponding DRT spectra in the frequency range of 10^−2^–10^8^ Hz are shown for (**d**) fresh cells, (**e**) after formation, and (**f**) after 1000 cycles at 5 A g^−1^. The inset panels displayed using a logarithmic intensity scale.

Figure 6 summarizes the cycling-dependent impedance evolution and interfacial kinetic behavior of TNO electrodes in EDF, NOMAF, and TFMAF electrolytes. Figure 6a–c show the Nyquist responses at different electrochemical states (fresh, after formation, and after 1000 cycles at 5 A g^−1^), with insets presenting the full spectra and the main panels highlighting the low-impedance region. The corresponding DRT spectra (Figure 6d–f) provide mechanistic insight into resistance evolution. Based on widely accepted interpretations [64,65,66,67], the relaxation spectrum is divided into five regions (I–V), from high to low frequency. Region I (10^4^–10^7^ Hz) reflects ultrafast instrumental or inductive responses [68]. Region II (10^2^–10^4^ Hz) is associated with electronic/contact-related processes. Region III (10^1^–10^2^ Hz) corresponds to Li^+^ transport across the interphase layer. Region IV (~10^0^–10^1^ Hz) is attributed to interfacial charge-transfer reactions, while Region V (~10^−1^–10^0^ Hz) represents diffusion-related polarization. Although these assignments follow established interpretations, partial overlap between processes cannot be excluded.

In the fresh state (Figure 6a,d), clear electrolyte-dependent behavior is observed. EDF exhibits the lowest overall impedance, whereas TFMAF and NOMAF show higher responses. TFMAF displays a longer low-frequency tail, indicating stronger diffusion-related polarization, while NOMAF shows a larger intermediate-frequency arc, suggesting more pronounced charge-transfer resistance. DRT analysis confirms that Regions IV and V dominate the initial impedance, indicating that charge transfer and diffusion processes are the primary kinetic limitations prior to activation. NOMAF exhibits the highest peak intensity in both Regions IV and V, followed by TFMAF and EDF, indicating stronger initial interfacial polarization and diffusion resistance in the flame-retardant systems. Contributions from Regions II and III are minor, and Region I shows negligible differences, confirming that initial variations are governed primarily by interfacial and diffusion processes. After formation (Figure 6b,e), the overall impedance markedly decreases, reflecting the formation of an electrochemically stable interphase that facilitates Li^+^ transport and reduces charge-transfer resistance. Correspondingly, DRT intensities in Regions III–V are significantly reduced. The reduction is most pronounced in Region V, accompanied by a decrease in Region IV, indicating alleviated diffusion polarization and interfacial resistance. Despite this improvement, electrolyte-dependent differences persist: EDF shows the lowest overall response, NOMAF the highest, and TFMAF remains intermediate. Notably, NOMAF and TFMAF retain stronger responses than EDF in Regions IV and V, as well as slightly higher contributions in Regions II and III, indicating residual kinetic limitations in the flame-retardant systems. Region I remains unchanged, consistent with its non-electrochemical origin. After prolonged cycling (Figure 6c,f), distinct electrolyte-dependent differences remain. In Region V, NOMAF exhibits the highest peak intensity, followed by EDF, while TFMAF shows the lowest, indicating that TFMAF most effectively suppresses diffusion-related resistance, whereas NOMAF retains the strongest diffusion polarization. In Region IV, EDF exhibits the most prominent residual response, indicating more pronounced charge-transfer limitations after long-term cycling. In Region III, NOMAF shows a broader response, suggesting persistent interphase transport resistance. Similarly, NOMAF exhibits the strongest response in Region II, indicating residual contact-related resistance. Region I shows no significant differences, confirming its weak dependence on electrolyte chemistry. Notably, across all states, the Region IV peak of EDF appears at lower frequencies than those of NOMAF and TFMAF, indicating a distinct interfacial relaxation time constant.

Overall, the impedance evolution of TNO electrodes is strongly electrolyte-dependent. EDF exhibits relatively slower charge-transfer kinetics (lower-frequency Region IV peak) but maintains lower overall impedance before prolonged cycling. In contrast, NOMAF shows increased diffusion, interphase, and contact-related resistances after long-term cycling. TFMAF exhibits the lowest overall impedance after extended cycling, indicating more stable kinetic behavior. These results highlight distinct trade-offs among the electrolytes and emphasize the critical role of electrolyte chemistry in governing interfacial and transport properties during cycling.

**Figure 7:**
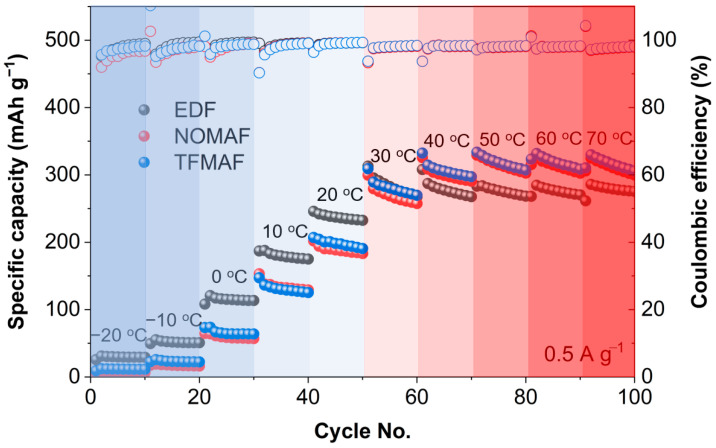
Specific discharge capacity and Coulombic efficiency of EDF, NOMAF, and TFMAF electrodes measured at a current density of 0.5 A g^−1^ within a voltage window of 0.01–3.0 V over a temperature range from −20 to 70 °C. Ten charge–discharge cycles were conducted at each temperature.

Figure 7 highlights the temperature-dependent electrochemical responses of the electrolyte systems over a wide operating range from −20 to 70 °C. In the sub-ambient region (<30 °C), EDF exhibits a gradual decrease in specific capacity with decreasing temperature, consistent with increased kinetic polarization arising from reduced ionic conductivity and slower charge-transfer processes. In contrast, the flame-retardant electrolytes (NOMAF and TFMAF) display more pronounced capacity variations under identical conditions, indicating stronger sensitivity of ion transport and desolvation kinetics to low-temperature constraints. Notably, within each low-temperature segment, only minimal capacity decay is observed during repeated cycling, suggesting that performance limitation in this regime primarily originates from transport-controlled kinetics rather than progressive interfacial degradation. Above 30 °C, the electrochemical behavior becomes increasingly temperature-dependent. EDF maintains relatively stable capacity with moderate thermal activation, whereas NOMAF and TFMAF exhibit progressive capacity enhancement with increasing temperature, followed by partial saturation or slight decline at 60–70 °C. This trend reflects reduced polarization resulting from improved ionic transport and accelerated interfacial kinetics at elevated temperatures, while subtle intra-segment capacity decay at higher temperatures suggests increasing contributions from thermally activated interfacial evolution and kinetic instability. Importantly, the Coulombic efficiency remains close to unity across the entire temperature range, indicating highly reversible Li^+^ storage and the absence of severe irreversible lithium consumption. Therefore, the observed temperature-dependent capacity variation is primarily governed by changes in transport and interfacial kinetics rather than sustained parasitic side reactions. The larger temperature-induced capacity fluctuations observed in the flame-retardant electrolytes are thus attributed not to continuous thermal activation, but to their greater sensitivity of transport and interfacial processes to temperature variation compared with the conventional carbonate-based system.

To further quantify these temperature-dependent kinetics, Arrhenius plots were constructed using the average specific capacities at each temperature (Figure S11). Due to the inverse temperature axis (1000/T), the left side corresponds to the high-temperature region (30–70 °C), while the right side represents the low-temperature region (30 to −20 °C). In the low-temperature region, relatively high activation energies of 29.5, 47.5, and 41.6 kJ mol^−1^ were obtained for EDF, NOMAF, and TFMAF, respectively, indicating that the electrochemical performance is strongly governed by thermally activated ion transport and interfacial charge-transfer processes. This suggests that ion desolvation and diffusion limitations dominate under sub-ambient conditions. In contrast, the high-temperature region exhibits near-zero or slightly negative apparent activation energies, indicating a deviation from ideal Arrhenius behavior and reduced temperature dependence of the electrochemical response. In this regime, transport and interfacial limitations are largely alleviated, and further temperature increases do not result in significant improvements in overall electrochemical performance.

**Figure 8:**
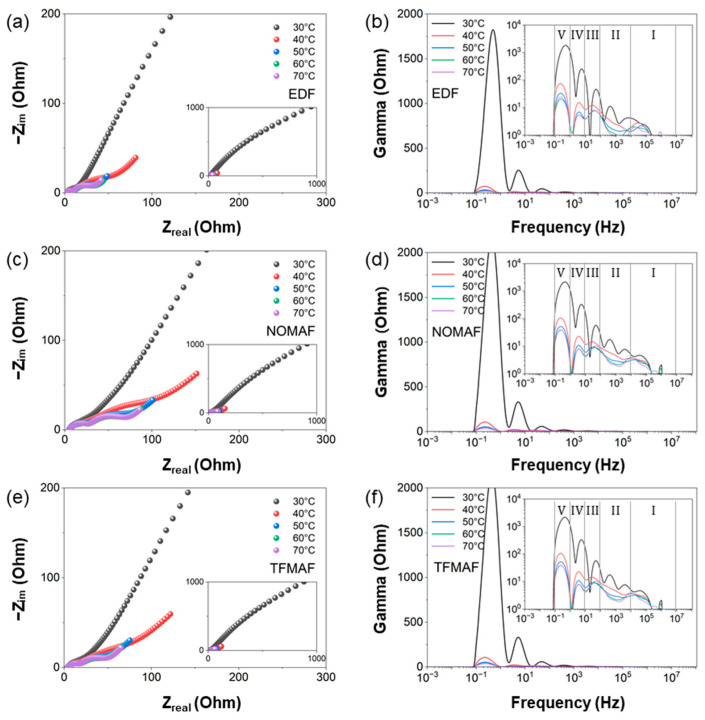
Electrochemical impedance spectroscopy (EIS) Nyquist plots and the corresponding distribution of relaxation time (DRT) analyses for cells with different electrolytes measured at elevated temperatures (30 to 70 °C) within the 0.01–3.0 V cycling range. Nyquist plots for (**a**) EDF, (**b**) NOMAF, and (**c**) TFMAF, with insets showing magnified views of the high-frequency region. Corresponding 2D DRT spectra (10^−3^–10^8^ Hz) for (**d**) EDF, (**e**) NOMAF, and (**f**) TFMAF. The inset panels use a logarithmic intensity scale.

**Figure 9:**
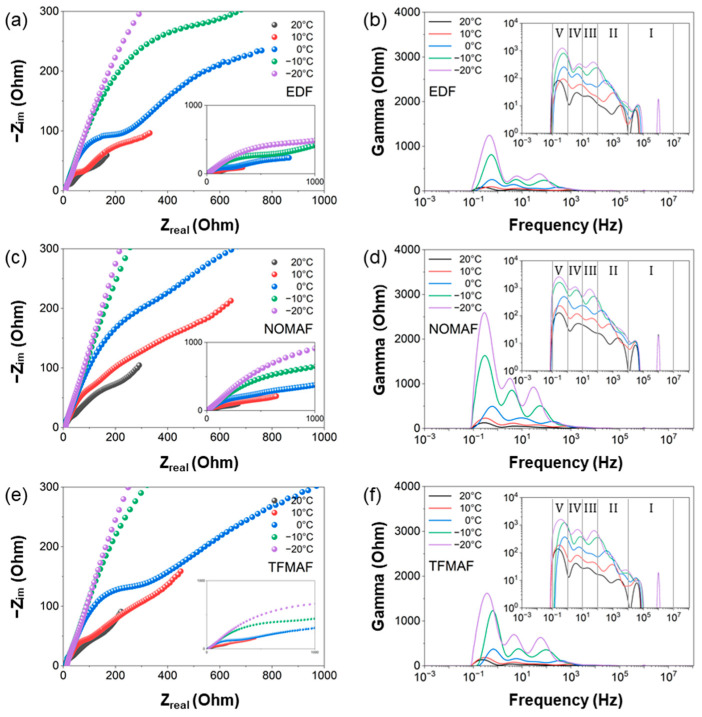
Electrochemical impedance spectroscopy (EIS) Nyquist plots and corresponding distribution of relaxation time (DRT) analyses for cells with different electrolytes, measured at low temperatures (20 to −20 °C) within a voltage window of 0.01–3.0 V. Nyquist plots for (**a**) EDF, (**b**) NOMAF and (**c**) TFMAF. The insets in (**a**–**c**) provide magnified views of the high-frequency region. Corresponding 2D DRT spectra (10^−3^–10^8^ Hz) for (**d**) EDF, (**e**) NOMAF, and (**f**) TFMAF. The inset panels display a logarithmic intensity scale.

To further elucidate the kinetic origin of the temperature-dependent behavior observed in Figure 7, temperature-resolved EIS and DRT analyses were conducted in both low- and high-temperature regimes, as presented in Figure 8 and Figure 9. Figure 8 summarizes the temperature-dependent impedance evolution of the three electrolyte systems (EDF, NOMAF, and TFMAF) within the elevated temperature range of 30–70 °C, corresponding to the high-temperature regime identified in Figure 7. Figure 8a–c present the Nyquist plots measured at different temperatures. Insets display the full impedance spectra, whereas the main panels highlight magnified views of the low-impedance region to enable clearer comparison of temperature-induced variations. For impedance measurements, cells were initially cycled for 10 cycles at 0.5 A g^−1^ and 30 °C prior to EIS acquisition. At each subsequent temperature step, the cells were equilibrated at the target temperature for 2 h, followed by an additional 10 conditioning cycles before impedance measurement to ensure stable electrochemical states. With increasing temperature, all Nyquist plots progressively contract toward the lower-left region, indicating systematic reductions in both electrolyte resistance and interfacial impedance components. The shrinkage of the high- and mid-frequency semicircles reflects decreased ohmic resistance together with improved interfacial charge-transfer processes, confirming that elevated temperature enhances ionic conductivity and accelerates interfacial reaction kinetics. The corresponding DRT spectra (Figure 8d–f) further resolve the temperature-dependent evolution of relaxation processes across distinct frequency domains. As temperature increases from 30 to 70 °C, the overall DRT peak intensities markedly decrease for all electrolytes, with the most pronounced suppression observed in the low-frequency Region V. This behavior indicates that impedance at lower temperature is largely governed by mass-transport limitations, whereas elevated temperature significantly promotes Li^+^ diffusion and mitigates diffusion-induced polarization. Concurrent reductions in Regions III and IV further suggest accelerated interphase transport and enhanced charge-transfer kinetics at elevated temperatures. Comparison among the electrolytes reveals that NOMAF and TFMAF consistently exhibit higher peak intensities than EDF across the temperature range, indicating relatively stronger interfacial and diffusion-related polarization in the flame-retardant systems. This behavior is likely associated with differences in interphase properties and ionic transport characteristics arising from electrolyte chemistry. Additionally, the Region III peak shifts toward lower frequency above ~40 °C for all electrolytes. Because the characteristic relaxation time follows τ = 1/(2πf), this shift indicates an increase in the effective relaxation time, which may arise from temperature-induced interphase evolution and altered relaxation dynamics at the electrode/electrolyte interface.

Figure 9 summarizes the temperature-dependent impedance evolution of the three electrolytes (EDF, NOMAF, and TFMAF) within the sub-ambient temperature range (−20 to 20 °C). Figure 9a–c presents the EIS Nyquist responses measured under low-temperature conditions corresponding to the sub-ambient regime identified in Figure 7. Insets display magnified views of the high- to mid-frequency region, enabling clearer comparison of temperature-induced variations in interfacial impedance. As the temperature decreases from 20 to −20 °C, all Nyquist plots progressively expand toward higher real and imaginary impedance values, indicating substantial increases in both bulk electrolyte resistance and interfacial polarization. The pronounced enlargement of the mid-frequency semicircle reflects a significant increase in charge-transfer resistance, whereas the elongation and steepening of the low-frequency tail indicate increasingly hindered ion diffusion. Collectively, these observations confirm that reduced temperature suppresses ionic conductivity and substantially slows interfacial electrochemical kinetics.

The corresponding DRT spectra (Figure 9d–f) provide mechanistic resolution of these temperature-dependent changes. Consistent with the relaxation-domain classification established in Figure 8 (Regions I–V), the most pronounced variation occurs in the low-frequency Region V, where peak intensities increase dramatically with decreasing temperature. This behavior indicates that mass-transport processes—including solid-state Li^+^ diffusion within TNO and ionic transport through the porous electrode—become the dominant kinetic limitation under sub-ambient conditions. Simultaneously, significant peak amplification is observed in Regions IV and III, corresponding, respectively, to interfacial charge-transfer reactions and Li^+^ migration across the SEI/interphase layer. The strong enhancement of these relaxation features at −10 and −20 °C indicates increasingly sluggish desolvation and interphase transport processes as thermal energy decreases. These results demonstrate that low-temperature performance degradation originates from coupled limitations in both interfacial reaction kinetics and ion transport rather than from structural electrode degradation. Comparison among the electrolytes reveals that NOMAF and TFMAF exhibit more pronounced peak growth than EDF across Regions III–V, indicating greater sensitivity of interfacial and diffusion processes to low-temperature constraints in flame-retardant electrolyte systems. This trend is consistent with higher Li^+^ desolvation barriers and reduced ionic mobility associated with fluorinated solvation structures. Moreover, slight shifts in the Region III–IV peaks toward higher frequency at intermediate temperatures (e.g., 10 °C) suggest partial kinetic compensation arising from moderate interphase stabilization, whereas the pronounced shift toward lower frequency at −20 °C reflects a substantial increase in the effective relaxation time constant (τ), consistent with severely hindered interfacial and diffusion dynamics.

Collectively, the combined Nyquist and DRT analyses demonstrate that sub-ambient operation induces transport-dominated polarization, in contrast to the diffusion-suppression behavior observed at elevated temperatures (Figure 8). These results confirm that electrolyte chemistry governs the temperature sensitivity of interfacial and diffusion kinetics, thereby determining low-temperature electrochemical accessibility in TNO electrodes.

**Figure 10:**
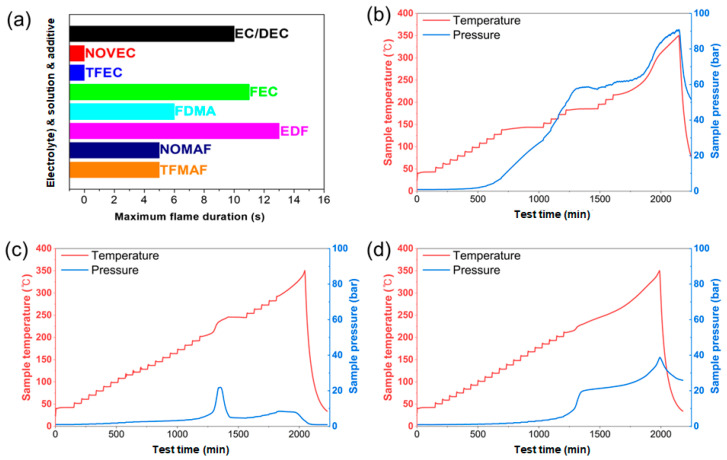
Flammability and thermal-safety evaluation of the electrolyte systems. (**a**) Self-extinguishing time (SET) results comparing the maximum flame duration of individual solvents and additives (EC/DEC, TFEC, NOVEC, FEC, and FDMA) together with the corresponding electrolyte formulations (EDF, NOMAF, and TFMAF). (**b**–**d**) Accelerating rate calorimetry (ARC) profiles showing the evolution of sample temperature (red) and internal pressure (blue) as a function of test time for (**b**) EDF, (**c**) NOMAF, and (**d**) TFMAF electrolytes.

Figure 10 presents a comprehensive safety evaluation of the electrolyte systems through flammability and thermal-abuse analyses. Figure 10a summarizes the self-extinguishing time (SET) results by comparing the maximum flame duration of individual solvents/additives together with their corresponding electrolyte formulations. Carbonate-based EC/DEC and the EDF electrolyte exhibit markedly prolonged flame durations, indicating high intrinsic flammability and sustained combustion behavior. In contrast, fluorinated solvents such as TFEC and NOVEC display negligible flame persistence, reflecting strong flame-suppression capability. Incorporation of phosphate-containing FDMA and fluorinated components in the mixed electrolytes (NOMAF and TFMAF) substantially reduces flame duration relative to EDF, demonstrating pronounced self-extinguishing characteristics. These observations indicate that fluorinated and phosphate-based constituents effectively interrupt combustion propagation and suppress flame sustainability. Representative SET photographs in Figure S12 visually corroborate the quantitative results shown in Figure 10a. ECDEC, FEC, and EDF exhibit sustained burning after ignition, indicating high flammability. In contrast, NOVEC and TFEC extinguish immediately upon removal of the ignition source, consistent with non-flammable behavior. FDMA, NOMAF, and TFMAF exhibit only short-lived flames and self-extinguish within a few seconds, indicating that they have flame-retardant characteristics. Overall, the incorporation of fluorinated, non-flammable components effectively suppresses flame propagation and enhances the fire safety of the electrolyte systems.

To further understand the electrolyte-dependent cycling behavior, we measured the room-temperature ionic conductivity and Li^+^ transference number of EDF, NOMAF, and TFMAF using SS|SS blocking cells and potentiostatic polarization. The results (Figures S13 and S14 and Table S4) show that TFMAF has the highest ionic conductivity and Li^+^ transference number, contributing to its improved rate capability and cycling stability compared with NOMAF. However, EDF still exhibits the best overall electrochemical performance despite its slightly lower ionic conductivity and Li^+^ transference number, indicating that bulk transport properties alone do not govern cell behavior. Instead, interfacial charge-transfer kinetics, SEI/interphase stability, and electrolyte–electrode compatibility also play key roles, as supported by the EIS/DRT and XPS analyses.

Figure 10b–d further compare the thermal stability of EDF, NOMAF, and TFMAF electrolytes using accelerating rate calorimetry (ARC), while the corresponding self-heating profiles are summarized in Figure S15 as plots of sample temperature versus the rate of temperature change. As shown in Figure S10a, EDF exhibits the broadest and most continuous self-heating region above 200 °C, with the rate of temperature rise increasing markedly over the range of 220–320 °C. This behavior is consistent with the slope observed above 220 °C in Figure 10b and supports the occurrence of exothermic decomposition processes. In Figure 10b, EDF exhibits two distinct plateau regions in the temperature profile, indicating multi-step exothermic decomposition processes. Self-heating begins at approximately 200 °C. Although the self-heating rate initially remains below the ARC thermal runaway criterion, continuous decomposition reactions promote sustained gas evolution, ultimately leading to a sharp pressure increase to ~90 bar. This behavior indicates substantial heat release accompanied by significant gas generation, representing the highest thermal hazard among the tested electrolytes. In contrast, NOMAF (Figure 10c) exhibits the most restrained thermal behavior. A plateau region near ~200 °C suggests weak self-heating at intermediate temperatures, while the maximum pressure increases only to ~20 bar. Although further temperature increases occur above ~250 °C, they are not accompanied by a proportional pressure rise, indicating limited formation of volatile decomposition products. Consistently, the ARC profile in Figure S10b shows that, for NOMAF, the high-temperature self-heating region is less extensive than that of EDF. The more dispersed and less accelerated rate response indicates that thermally triggered reactions are effectively suppressed. TFMAF (Figure 10d) exhibits a moderated thermal response compared with EDF, although it remains slightly less suppressed than NOMAF. Noticeable self-heating begins near ~200 °C, followed by a moderate pressure increase to approximately 40 bar. The reduced peak pressure relative to EDF suggests suppressed gas evolution and attenuated exothermic reactions due to the flame-retardant electrolyte composition. As shown in Figure S10c, TFMAF displays a more evident self-heating trajectory than NOMAF in the range of 220–330 °C, but its rate increase remains less extensive than that of EDF, indicating an intermediate level of thermal reactivity. Collectively, the ARC results demonstrate that the incorporation of fluorinated and phosphate-based flame-retardant components significantly mitigates both exothermic intensity and gas generation during thermal abuse. The self-heating rate profiles in Figure S11 further support this conclusion, showing that EDF undergoes the most pronounced and sustained thermal acceleration, whereas NOMAF exhibits the weakest self-heating response and TFMAF remains intermediate. Compared with the carbonate-based EDF electrolyte, the flame-retardant systems exhibit delayed self-heating onset and substantially reduced pressure accumulation, indicating improved resistance to thermal runaway.

Between the two flame-retardant systems, NOMAF exhibits superior thermal stability from an ARC perspective. Specifically, NOMAF shows a significantly lower maximum pressure (~20 bar) than TFMAF (~40 bar), indicating more effective suppression of gas evolution. In addition, the weaker coupling between temperature rise and pressure buildup suggests that NOMAF more effectively limits the formation of volatile decomposition products. In contrast, TFMAF, while still improved relative to EDF, exhibits a more pronounced pressure increase, implying comparatively higher gas generation and exothermic activity. Therefore, NOMAF provides the most stable thermal response among the tested electrolytes, offering enhanced safety under thermal abuse conditions [69].

## 4. Conclusions

This work systematically investigates the electrochemical performance, interfacial evolution, temperature-dependent kinetics, and thermal safety of TiNb_2_O_7_ nanofiber (TNO NF) anodes in conventional carbonate and flame-retardant electrolyte systems. Although TFMAF exhibits slightly better rate capability and long-term cycling stability than NOMAF, NOMAF demonstrated markedly enhanced thermal stability in both flammability and ARC tests by more effectively suppressing self-heating and pressure buildup. Importantly, NOMAF does not represent a system that is universally superior across all performance metrics. Rather, when safety, cost-effectiveness (considering the lower cost of NOVEC7200), and electrochemical performance within an acceptable range are comprehensively considered, NOMAF emerges as a practically viable electrolyte candidate. These results demonstrate that flame-retardant electrolytes can significantly improve battery safety without severely compromising electrochemical performance, providing important guidance for the development of safer lithium-ion batteries.

## Data Availability

The original contributions presented in this study are included in the article. Further inquiries can be directed to the corresponding author.

## Supplementary Materials

The following supporting information can be downloaded at: https://www.mdpi.com/article/10.3390/ma19091840/s1, Figure S1: XRD patterns of electrospun TNO samples under varying PVP contents and calcination temperatures, illustrating the phase evolution and crystallinity development. Figure S2: Galvanostatic charge–discharge (GCD) voltage profiles of TNO powder electrodes (a–c) and TNO nanofiber (NF) electrodes (d–f) measured in EDF, NOMAF, and TFMAF electrolytes at 0.1 A g−1 within the restricted voltage window of 1.0–3.0 V vs. Li/Li+ for the first three cycles. Figure S3: Cyclic voltammetry (CV) responses of TNO nanofiber (NF) anodes measured within a 1.0–3.0 V vs. Li/Li+ voltage window. (a–c) CV curves obtained during the initial three cycles at a scan rate of 0.1 mV s−1 in EDF, NOMAF, and TFMAF electrolytes, respectively, highlighting the electrochemical activation behavior in different electrolyte environments. Figure S4: (a–c) Cyclic voltammetry (CV) curves recorded at scan rates ranging from 0.1 to 5 mV s−1 for TNO nanofiber (NF) anodes in EDF, NOMAF, and TFMAF electrolytes, respectively, measured within a potential window of 1.0–3.0 V vs. Li/Li+. (d,e) Log–log plots of peak current as a function of scan rate with corresponding linear fittings used to determine the b-values for the oxidation (d) and reduction (e) processes. (f–h) Quantitative contributions of pseudocapacitive and diffusion-controlled charge-storage processes in EDF, NOMAF, and TFMAF electrolytes at scan rates from 0.1 to 5 mV s−1, evaluated using Dunn’s method. Figure S5: (a–c) Quantitative separation of pseudocapacitive and diffusion-controlled charge-storage contributions of TNO nanofiber (NF) electrodes in EDF, NOMAF, and TFMAF electrolytes at a scan rate of 3 mV s−1, evaluated using Dunn’s method within the full voltage window of 0.01–3.0 V vs. Li/Li+. (d–f) Corresponding deconvolution results obtained within the restricted voltage window of 1.0–3.0 V vs. Li/Li+. Figure S6: Rate capability comparison between TNO nanofiber (NF) and TNO powder anodes in different electrolytes under stepwise current densities ranging from 0.1 to 10 A g−1, followed by recovery at 0.1 A g−1. (a–c) Rate performance measured in EDF, NOMAF, and TFMAF electrolytes, respectively, within a voltage window of 0.01–3.0 V vs. Li/Li+. (d–f) Corresponding rate capability evaluated within a restricted voltage window of 1.0–3.0 V vs. Li/Li+. Figure S7: Long-term cycling performance and corresponding Coulombic efficiency of TNO nanofiber (NF) and powder anodes in EDF, NOMAF, and TFMAF electrolytes over 1000 cycles at a current density of 5 A g−1. (a–c) Cycling performance and Coulombic efficiency measured within a voltage window of 0.01–3.0 V vs. Li/Li+ in EDF, NOMAF, and TFMAF electrolytes, respectively. (d–f) Cycling performance and Coulombic efficiency measured within a restricted voltage window of 1.0–3.0 V vs. Li/Li+ in EDF, NOMAF, and TFMAF electrolytes, respectively. Figure S8: SEM images of TNO NF electrodes with different electrolytes after formation and prolonged cycling. (a–c) Morphologies after formation in (a) EDF, (b) NOMAF, and (c) TFMAF electrolytes. (d–f) Morphologies after 1000 cycles at high current density in (d) EDF, (e) NOMAF, and (f) TFMAF electrolytes. Figure S9: X-ray diffraction (XRD) analysis of the pristine TNO electrode and TNO electrodes after formation cycling at 0.1 A g−1 in EDF, NOMAF, and TFMAF electrolytes, collected over 2θ ranges of (a) 0–40° and (b) 40–70°. Figure S10: X-ray photoelectron spectroscopy (XPS) analysis [70,71,72,73,74,75,76,77,78] of the bare TNO electrode and TNO electrodes after two formation cycles in EDF, NOMAF, and TFMAF electrolytes. (a) Survey spectra and high-resolution spectra of (b) C 1s, (c) O 1s, (d) F 1s, (e) N 1s, (f) Ti 2p, (g) Nb 3d, (h) S 2p, (i) P 2p, and (j) Li 1s. Figure S11: Arrhenius plots of ln(specific capacity) as a function of 1000/T for EDF, NOMAF, and TFMAF electrolytes. The temperature range is divided into high-temperature (30–70 °C, left-hand side) and low-temperature (30 to –20 °C, right-hand side) regimes based on the inverse temperature axis (1000/T). Linear fitting is performed separately for each regime to extract the apparent activation energies (Ea). Figure S12: Representative time-sequenced images recorded during the self-extinguishing time (SET) test for individual solvents and corresponding electrolyte formulations. The image sequences illustrate ignition behavior, flame propagation, and extinction characteristics during combustion. Carbonate-based solvents and the EDF electrolyte exhibit sustained flame propagation, whereas fluorinated solvents and phosphate-containing electrolyte systems show rapid flame suppression or self-extinguishing behavior, demonstrating enhanced flame-retardant characteristics. Figure S13: Nyquist plots of SS|SS cells at room temperature for ECDEC, NOMAF, and TFMAF electrolytes. Figure S14: Lithium-ion transference number (tLi+) measurement for EDF, NOMAF, and TFMAF Electrolytes. The current response of the electrolytes during the DC polarization test is shown for (a) EDF, (b) NOMAF, and (c) TFMAF electrolytes. The insets in each panel show the corresponding EIS spectra before and after polarization. Figure S15: Accelerating rate calorimetry (ARC) profiles of (a) EDF, (b) NOMAF, and (c) TFMAF electrolytes, plotted as sample temperature (°C) versus the rate of temperature change (°C min−1). Table S1: Quantitative separation of irreversible loss and reversible capacity gain in different voltage windows. Table S2: Second-cycle discharge capacities of EDF, NOMAF, and TFMAF measured at stepwise current densities (0.1, 0.5, 1, 3, 5, and 10 A g−1) under two voltage windows (0.01–3.0 V and 1.0–3.0 V), along with the corresponding capacity retention values upon returning to 0.1 A g−1. Table S3: Discharge capacities measured at the 1st and 1000th cycles and the corresponding capacity retention values of TNO nanofiber (NF) anodes in EDF, NOMAF, and TFMAF electrolytes at a high current density of 5 A g−1 under two voltage windows (0.01–3.0 V and 1.0–3.0 V vs. Li/Li+). The data summarize long-term cycling performance and highlight the influence of electrolyte composition and operating voltage window on capacity retention behavior. Table S4: Room-temperature ionic conductivity, bulk resistance (R), separator thickness (d), and effective area (A) for EDF, NOMAF, and TFMAF electrolytes.

## Abbreviations

The following abbreviations are used in this manuscript:

TNO	TiNb_2_O_7_
EC	Ethylene carbonate
DEC	Diethyl carbonate
TFEC	Bis(2,2,2-trifluoroethyl) carbonate
NOVEC	Methyl nonafluorobutyl ether
FDMA	2,2,2-trifluoro-N,N-dimethylacetamide
LIBs	Lithium-ion Batteries
NFs	Nanofibers
EDF	1 M LiPF_6_ in EC/DEC = 1:1, *v*/*v*, with 10 wt% FEC
TFMAF	1 M LiTFSI in TFEC/FDMA = 1:1, *v*/*v*, with 10 wt% FEC
NOMAF	1 M LiTFSI in NOVEC7200/FDMA = 1:1, *v*/*v*, with 10 wt% FEC
ARC	Accelerating Rate Calorimetry
PVP	Poly(Vinyl Pyrrolidone)
CMC	Carboxymethyl Cellulose
SBR	Styrene-Butadiene Rubber
XRD	X-ray Diffraction
FE-SEM	Field-Emission Scanning Electron Microscopy
FEC	Fluoroethylene Carbonate
LiTFSI	Lithium bis(trifluoromethanesulfonyl)imide
SET	Self-Extinguishing Time
HWS	Heat–Wait–Search
PP-PE–PP	Polypropylene–Polyethylene–Polypropylene
GCD	Galvanostatic Charge–Discharge
CV	Cyclic Voltammetry
EIS	Electrochemical Impedance Spectroscopy
DRT	Distribution of Relaxation Times
SEI	Solid Electrolyte Interphase
