# Supplementary material for: Electrochemical Performance of TiNb2O7 Nanofibers for Lithium-Ion Battery Anodes Using Flame-Retardant Electrolytes
Source: Materials (Basel). 2026 Apr 29;19(9):1840. doi: 10.3390/ma19091840 (PMC13165023; doi:10.3390/ma19091840)
Supplement: Supplementary file 1 [file materials-19-01840-s001.zip › materials-4259451-supplementary.pdf]

Supplementary Materials

Electrochemical Performance of $TiNb_2O_7$ Nanofibers for Lithium-Ion Battery Anodes Using Flame-Retardant Electrolytes

Seongwon Go [1,†], Hong Chen [1,†], Seul Lee [1], Garim Lee [1], Hye Seon Yoon [1], Minseung Kang [1], and Chae-Ryong Cho [1,2,3*]

[1] Department of Nano Fusion Technology, Pusan National University, Busan 46241, Republic of Korea
[2] School of Transdisciplinary Engineering, Pusan National University, Busan 46241, Republic of Korea
[3] Department of Nano Energy Engineering, Pusan National University, Busan 46241, Republic of Korea
* Correspondence: crcho@pusan.ac.kr; Tel.: +82-51-510-6114
† These authors contributed equally to this work.

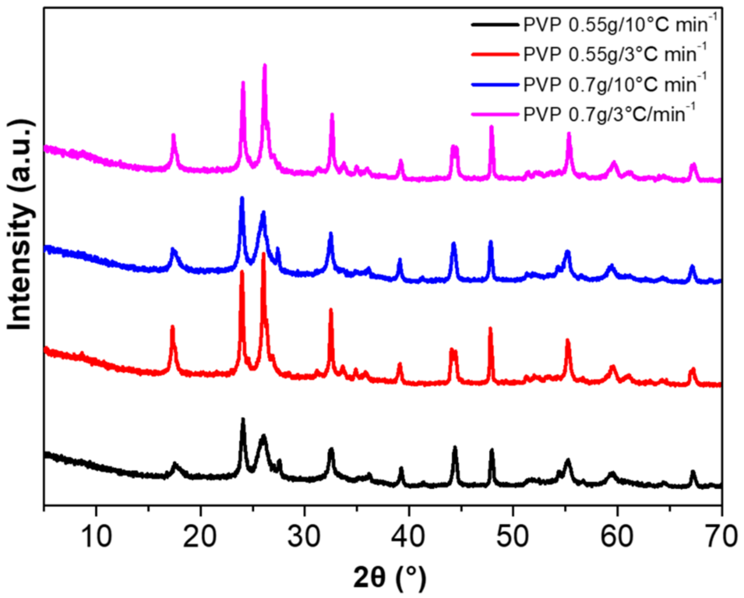

Figure S1. XRD patterns of electrospun TNO samples under varying PVP contents and calcination temperatures, illustrating the phase evolution and crystallinity development.

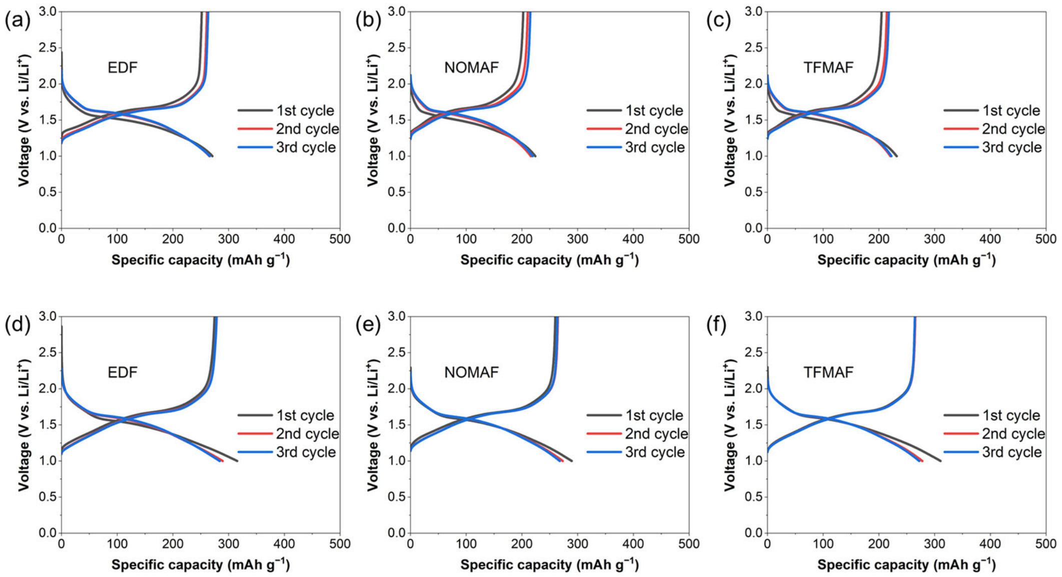

Figure S2. Galvanostatic charge–discharge (GCD) voltage profiles of TNO powder electrodes (a–c) and TNO nanofiber (NF) electrodes (d–f) measured in EDF, NOMAF, and TFMAF electrolytes at 0.1 A g^{-1} within the restricted voltage window of 1.0–3.0 V vs. Li/Li^{+} for the first three cycles.

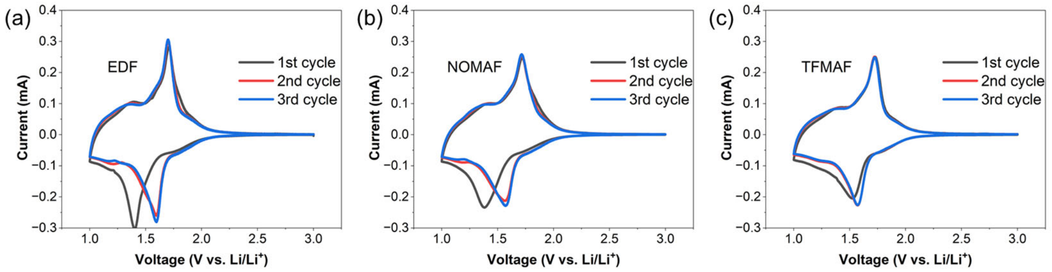

Figure S3. Cyclic voltammetry (CV) responses of TNO nanofiber (NF) anodes measured within a 1.0–3.0 V vs. Li/Li^+ voltage window. (a–c) CV curves obtained during the initial three cycles at a scan rate of 0.1 mV s^{-1} in EDF, NOMAF, and TFMAF electrolytes, respectively, highlighting the electrochemical activation behavior in different electrolyte environments.

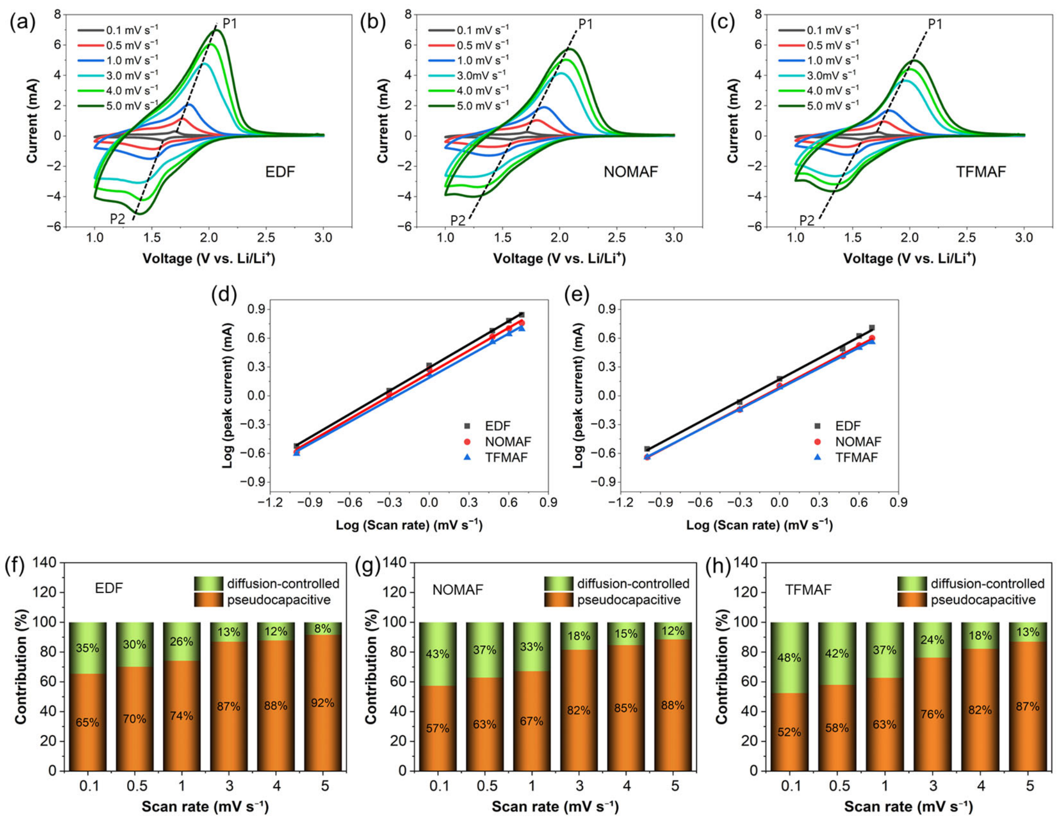

Figure S4. (a–c) Cyclic voltammetry (CV) curves recorded at scan rates ranging from 0.1 to 5.0 mV s^{-1} for TNO nanofiber (NF) anodes in EDF, NOMAF, and TFMAF electrolytes, respectively, measured within a potential window of 1.0–3.0 V vs. Li/Li^+. (d,e) Log–log plots of peak current as a function of scan rate together with corresponding *b*-values for the oxidation (P1) and reduction(P2) peaks, as marked in (a-c). (f–h) Quantitative contributions of pseudocapacitive and diffusion-controlled charge-storage processes in EDF, NOMAF, and TFMAF electrolytes at scan rates from 0.1 to 5.0 mV s^{-1}, evaluated using Dunn's method.

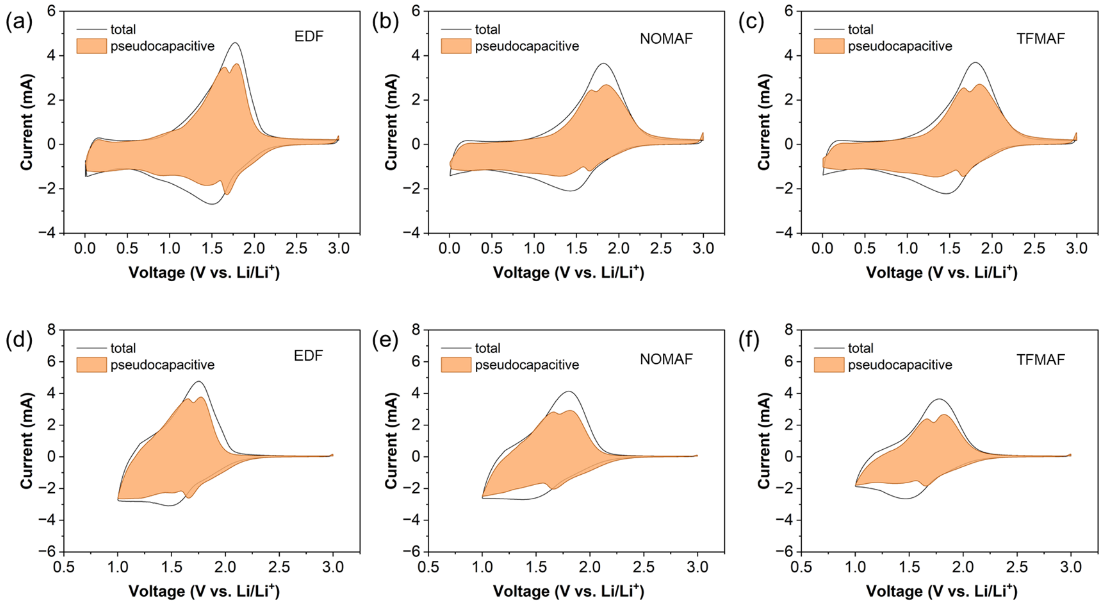

Figure S5. (a–c) Quantitative separation of pseudocapacitive and diffusion-controlled charge-storage contributions of TNO nanofiber (NF) electrodes in EDF, NOMAF, and TFMAF electrolytes at a scan rate of 3.0 mV s^{-1}, evaluated using Dunn's method within the full voltage window of 0.01–3.0 V vs. Li/Li^+. (d–f) Corresponding deconvolution results obtained within the restricted voltage window of 1.0–3.0 V vs. Li/Li^+.

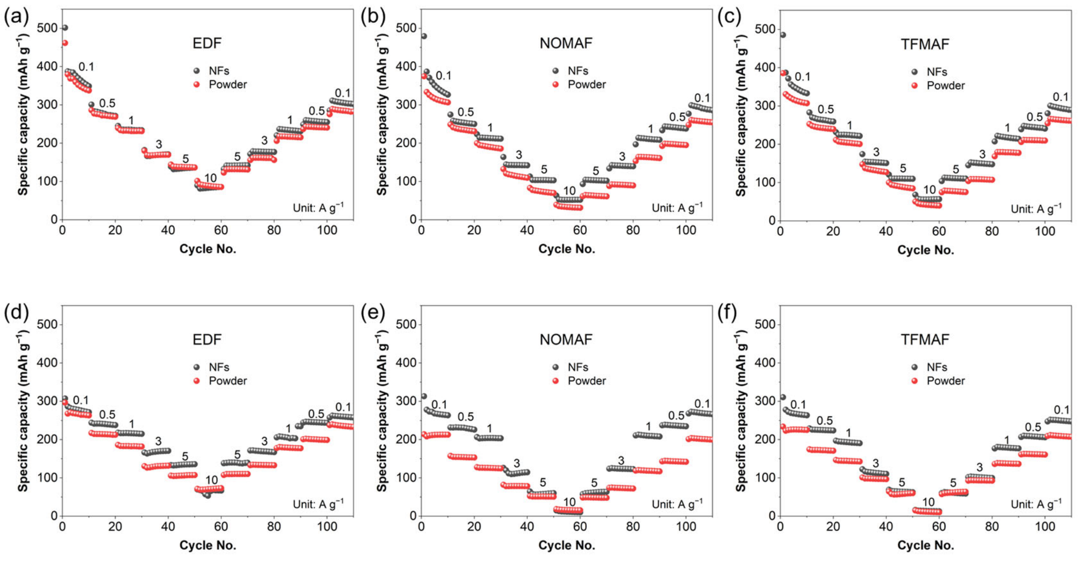

Figure S6. Rate capability comparison between TNO nanofiber (NF) and TNO powder anodes in different electrolytes under stepwise current densities ranging from 0.1 to 10 A g^{-1}, followed by recovery at 0.1 A g^{-1}. (a–c) Rate performance measured in EDF, NOMAF, and TFMAF electrolytes, respectively, within a voltage window of 0.01–3.0 V vs. Li/Li^+. (d–f) Corresponding rate capability evaluated within a restricted voltage window of 1.0–3.0 V vs. Li/Li^+.

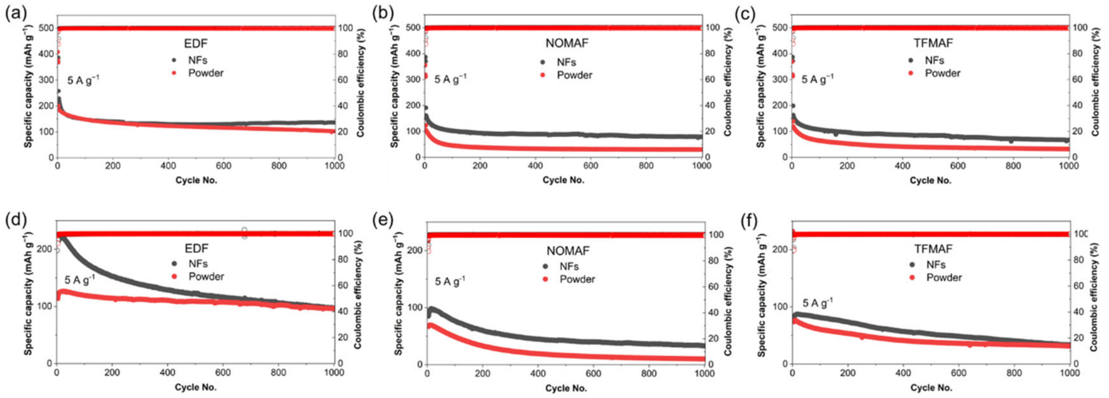

Figure S7. Long-term cycling performance and corresponding coulombic efficiency of TNO nanofiber (NF) and powder anodes in EDF, NOMAF, and TFMAF electrolytes over 1,000 cycles at a current density of 5 A g^{-1}. (a–c) Cycling performance and coulombic efficiency measured within a voltage window of 0.01–3.0 V vs. Li/Li^+ in EDF, NOMAF, and TFMAF electrolytes, respectively. (d–f) Cycling performance and coulombic efficiency measured within a restricted voltage window of 1.0–3.0 V vs. Li/Li^+ in EDF, NOMAF, and TFMAF electrolytes, respectively.

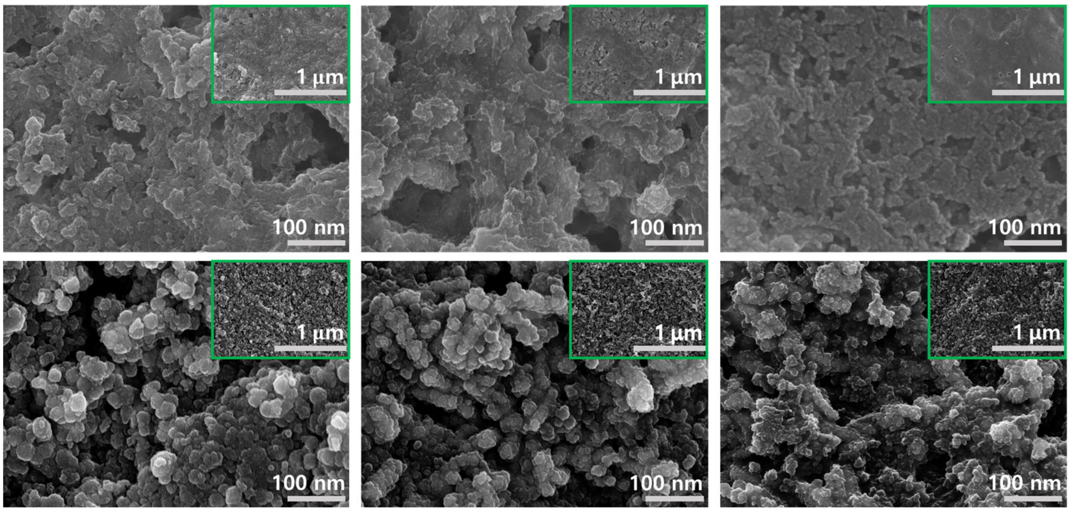

Figure S8. SEM images of TNO NF electrodes with different electrolytes after formation and prolonged cycling. (a–c) Morphologies after formation in (a) EDF, (b) NOMAF, and (c) TFMAF electrolytes. (d–f) Morphologies after 1,000 cycles at high current density in (d) EDF, (e) NOMAF, and (f) TFMAF electrolytes.

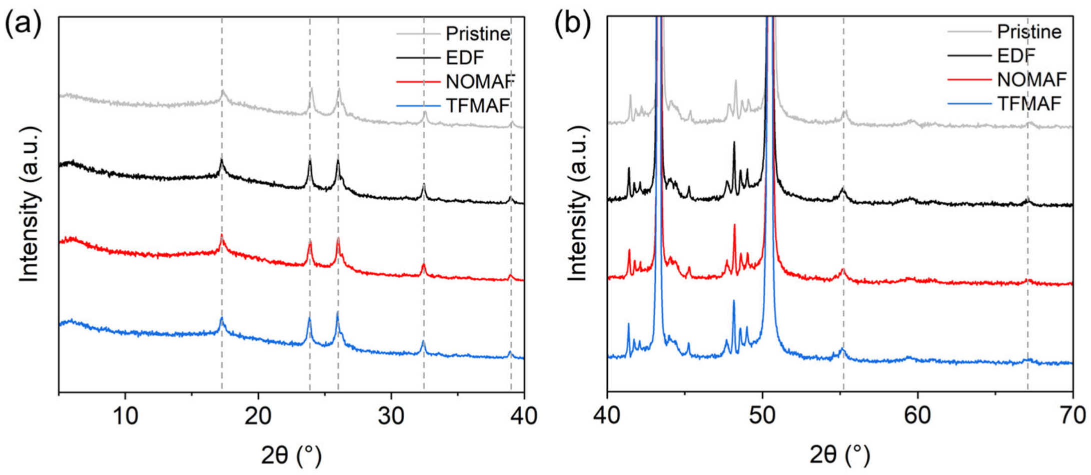

Figure S9. X-ray diffraction (XRD) analysis of the pristine TNO electrode and TNO electrodes after formation cycling at 0.1 A g^{-1} in EDF, NOMAF, and TFMAF electrolytes, collected over 2θ ranges of (a) 0–40° and (b) 40–70°.

To investigate whether structural changes occur during cycling in different electrolytes, XRD analysis was performed on the electrodes before cycling and after two cycles in EDF, NOMAF, and TFMAF electrolytes. Specifically, XRD patterns were obtained from the pristine electrode (prior to electrolyte exposure) and from electrodes after formation cycling in each electrolyte. The diffraction patterns exhibit nearly identical peak positions and comparable intensities across all samples, indicating that the bulk crystal structure of the electrode is well preserved regardless of the electrolyte type. In addition, no noticeable peak shifts, broadening, or emergence of new phases were observed, suggesting the absence of significant structural distortion or phase transformation during the initial cycling process.

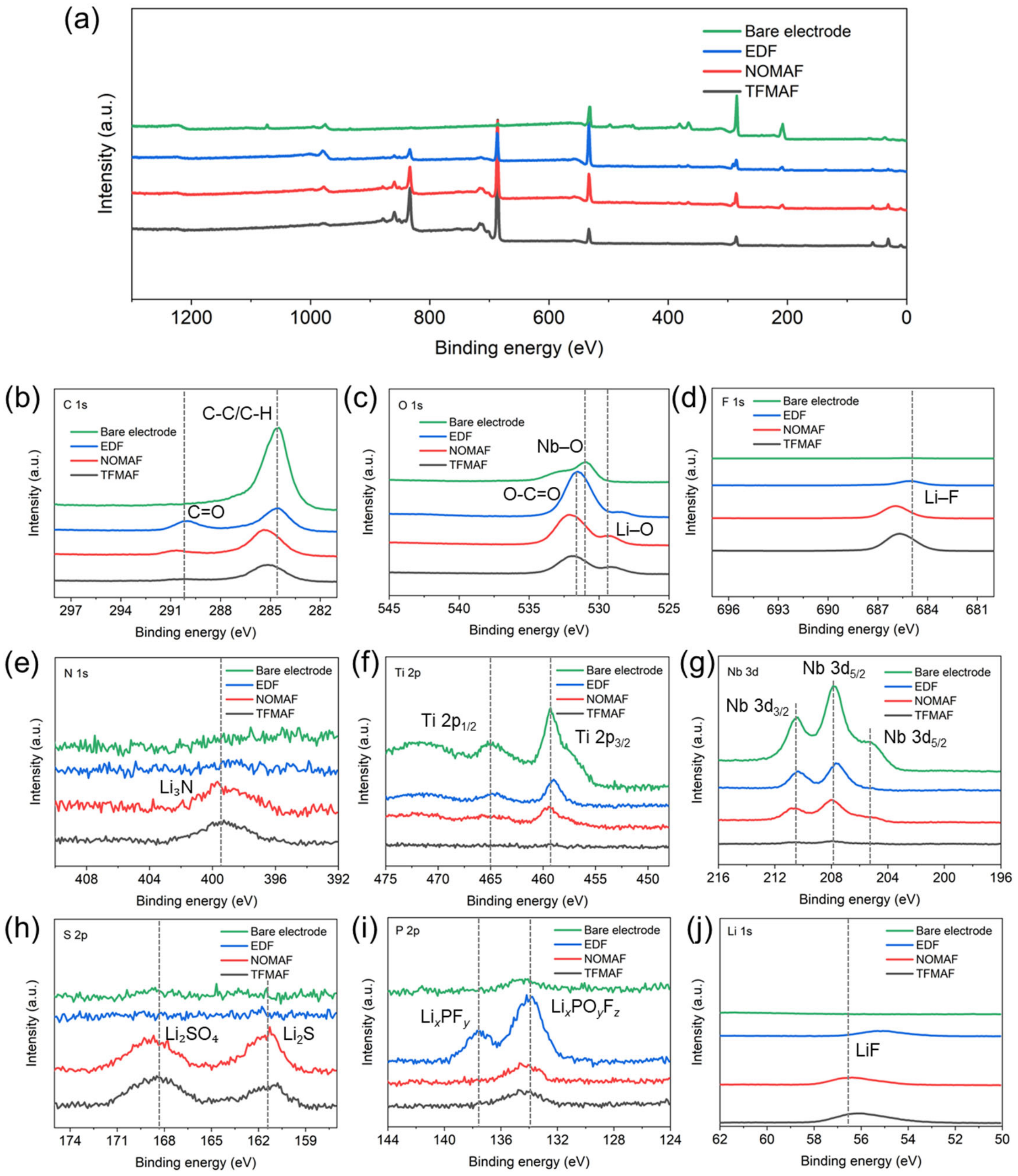

Figure S10. X-ray photoelectron spectroscopy (XPS) analysis of the bare TNO electrode and TNO electrodes after two formation cycles in EDF, NOMAF, and TFMAF electrolytes. (a) Survey spectra and high-resolution spectra of (b) C 1s, (c) O 1s, (d) F 1s, (e) N 1s, (f) Ti 2p, (g) Nb 3d, (h) S 2p, (i) P 2p, and (j) Li 1s.

The C 1s peaks at 284.5 and 290.0 eV are assigned to C–C/C–H and C=O species, respectively [1,2]. In the O 1s spectra, the Nb–O peak at 530.9 eV corresponds to the TNO lattice, while the O–C=O peak at 531.5–532.0 eV and the Li–O peak at 529.5 eV indicate carbonate-type SEI species, such as Li_2CO_3, lithium alkyl carbonates, and Li_2O [3]. The F 1s peak at 684.8 eV and the Li 1s peak at ~57.0 eV are attributed to LiF [4,5]. The N 1s signal at 399 eV is assigned to Li_3N-related species [6]. The Ti 2p spectra show Ti $2p_{3/2}$ and Ti $2p_{1/2}$

peaks at 459 and 465 eV, respectively, while the Nb 3d spectra display Nb $3d_{5/2}$ and Nb $3d_{3/2}$ peaks at 207.8 and 210.5 eV, respectively [7]. The S 2p peaks at 168.4/161.0 eV are assigned to Li_2SO_4/Li_2S [8], while the P 2p peaks at 134.0/137.6 eV correspond to $Li_xPO_yF_z$/ Li_xPF_y species [9]. The XPS results reveal clear electrolyte-dependent SEI/interphase chemistry on the cycled TNO electrodes. After cycling, the appearance of C=O, O–C=O, Li–O, and LiF signals in the C 1s, O 1s, F 1s, and Li 1s spectra confirms that electrolyte decomposition occurs during cycling, leading to the formation of a passivating interphase layer on the TNO surface.

In particular, the O–C=O signal in the O 1s spectrum is most pronounced for the EDF-cycled electrode, suggesting that the carbonate-based EDF electrolyte generates a larger amount of carbonate-type SEI species, such as Li_2CO_3 and lithium alkyl carbonates. These species may contribute to favorable interfacial contact and Li^+ transport, which is consistent with the higher reversible capacity of EDF observed during long-term cycling. However, the stronger carbonate-derived signal also implies more extensive decomposition of the carbonate-based electrolyte, which may be less favorable from a thermal safety perspective. The F 1s and Li 1s spectra further confirm the formation of LiF-containing interphase species after cycling. Notably, the LiF-related signal in the Li 1s spectrum is strongest for the TFMAF-cycled electrode, indicating that TFMAF promotes the formation of a more LiF-rich inorganic interphase. Because LiF is chemically stable and can help suppress continuous electrolyte decomposition, this LiF-rich interphase may be one of the interfacial factors contributing to the improved cycling stability of TFMAF compared with NOMAF among the flame-retardant electrolyte systems. In addition, the EDF-cycled electrode exhibits more pronounced phosphate-related signals in the P 2p spectrum, consistent with the decomposition of the $LiPF_6$-based electrolyte, whereas NOMAF and TFMAF show characteristic sulfur- and nitrogen-containing interphase species associated with LiTFSI-based systems. These results confirm that the surface chemistry of TNO evolves differently depending on the electrolyte composition.

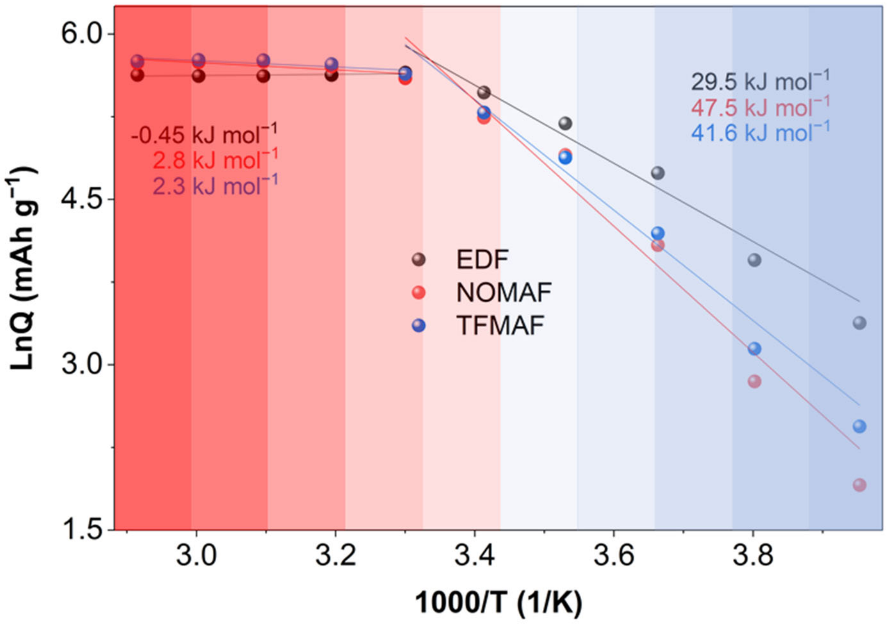

Figure S11. Arrhenius plots of ln(specific capacity) as a function of 1000/T for EDF, NOMAF, and TFMAF electrolytes. The temperature range is divided into high-temperature (30–70 °C, left-hand side) and low-temperature (30 to –20 °C, right-hand side) regimes based on the inverse temperature axis (1000/T). Linear fitting is performed separately for each regime to extract the apparent activation energies (E_a).

The Arrhenius analysis provides deeper mechanistic insight into the origin of the higher apparent activation energies observed for the flame-retardant (FR) electrolytes. Compared with the carbonate-based EDF system, both NOMAF and TFMAF exhibit significantly elevated activation energies in the low-temperature regime, indicating that their charge-storage kinetics are more strongly governed by thermally activated processes. This behavior can be primarily attributed to the distinct solvation structures and physicochemical properties of the fluorinated solvents and salts. First, the strong electron-withdrawing nature of fluorinated solvent molecules leads to weaker donor ability toward Li^+, resulting in more tightly bound and energetically stabilized solvation structures. Consequently, Li^+ desolvation at the electrode/electrolyte interface requires higher activation energy, particularly under sub-ambient conditions. In addition, the higher viscosity and lower ionic conductivity typically associated with fluorinated electrolyte systems further hinder ion transport, amplifying diffusion limitations at low temperatures. Second, the formation of interphases in FR electrolytes is often dominated by inorganic-rich components (e.g., LiF-derived species), which, although beneficial for thermal stability and safety, can introduce higher initial resistance and slower Li^+ transport across the interphase. This contributes to the increased activation barrier associated with interfacial charge-transfer processes, as reflected in the larger Arrhenius slopes.

In contrast, the carbonate-based EDF electrolyte, characterized by stronger solvation capability and lower viscosity, facilitates more efficient Li^+ transport and faster interfacial kinetics, resulting in a lower apparent activation energy. The relatively moderate activation energy observed for TFMAF compared with NOMAF further suggests that subtle differences in solvent composition and solvation structure can partially mitigate these kinetic limitations. At

elevated temperatures, the apparent activation energies approach near-zero or slightly negative values for all systems, indicating that thermal activation sufficiently overcomes desolvation and transport barriers. Under these conditions, kinetic limitations become less dominant, and the electrochemical response is no longer strongly temperature-dependent. This transition highlights that the performance gap between carbonate and FR electrolytes is primarily manifested under kinetically constrained, low-temperature conditions, whereas at higher temperatures, the intrinsic safety advantages of FR systems can be achieved without significant kinetic penalties.

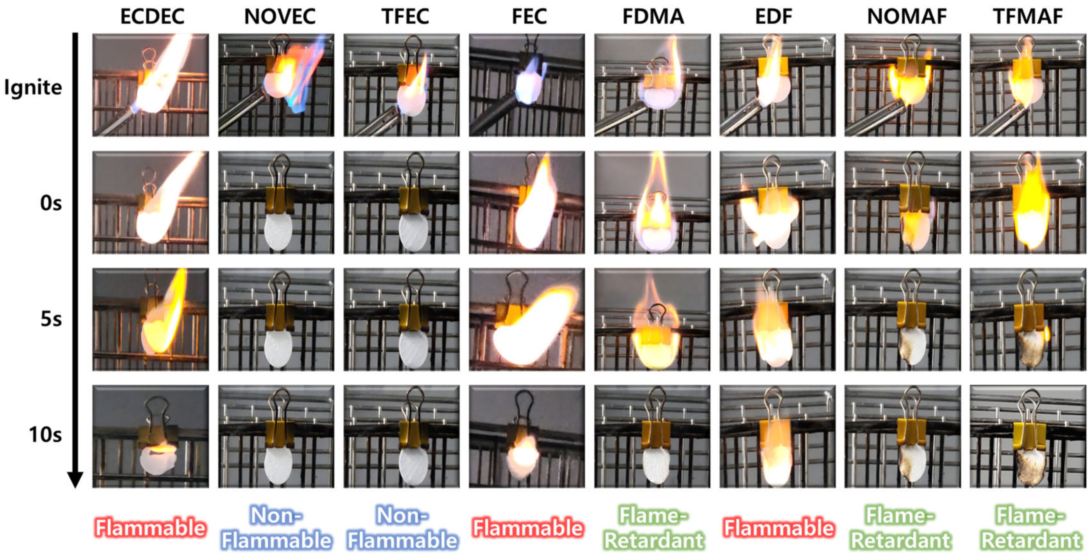

Figure S12. Representative time-sequenced images recorded during the self-extinguishing time (SET) test for individual solvents and corresponding electrolyte formulations. The image sequences illustrate ignition behavior, flame propagation, and extinction characteristics during combustion. Carbonate-based solvents and the EDF electrolyte exhibit sustained flame propagation, whereas fluorinated solvents and phosphate-containing electrolyte systems show rapid flame suppression or self-extinguishing behavior, demonstrating enhanced flame-retardant characteristics.

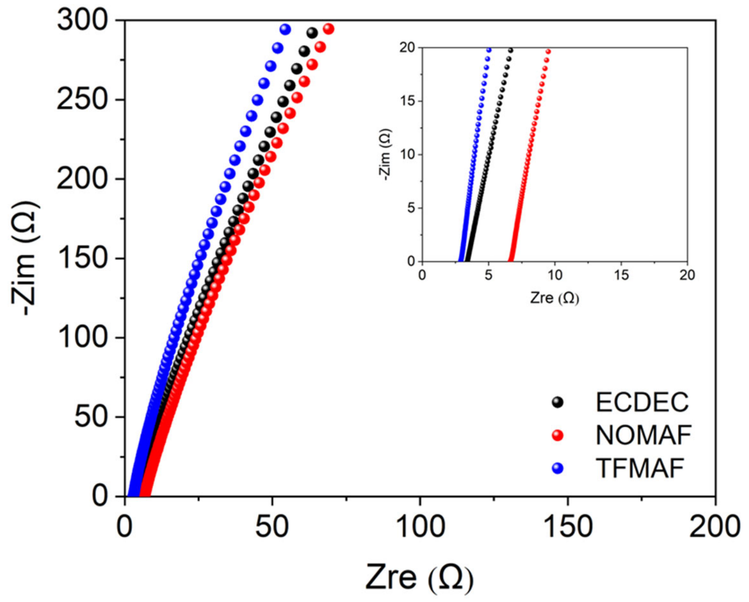

Figure S13. Nyquist plots of SS|SS cells measured at room temperature for EDF, NOMAF, and TFMAF electrolytes.

The measured ionic conductivities are 2.49 mS cm^{-1} (EDF), 1.27 mS cm^{-1} (NOMAF), and 2.92 mS cm^{-1} (TFMAF), indicating clear electrolyte-dependent differences in bulk ion transport. The relatively low ionic conductivity of NOMAF is consistent with its inferior rate capability. Although TFMAF exhibits slightly higher ionic conductivity than EDF, its overall electrochemical performance is limited by interfacial factors, highlighting that ionic conductivity alone does not govern rate capability. Instead, charge-transfer kinetics and interfacial stability play equally important roles.

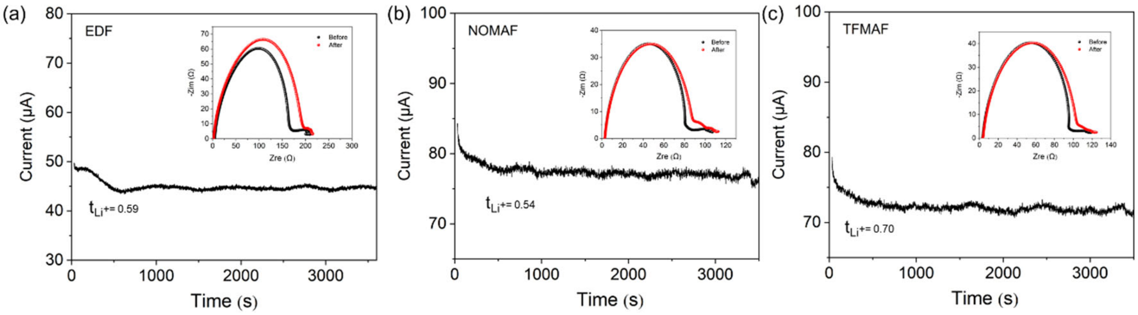

Figure S14. Lithium-ion transference number (t_{Li+}) measurement for EDF, NOMAF, and TFMAF Electrolytes. The current response of the electrolytes during the DC polarization test is shown for (a) EDF, (b) NOMAF, and (c) TFMAF electrolytes. The insets in each panel show the corresponding EIS spectra before and after polarization.

The calculated Li^+ transference numbers (t_{Li+}) are 0.59, 0.54, and 0.61 for EDF, NOMAF, and TFMAF, respectively. NOMAF exhibits the lowest t_{Li+} value, indicating that Li^+ contributes less effectively to the overall ionic current in this electrolyte, which may lead to stronger concentration polarization during high-rate cycling. This result is consistent with the lower rate capability and higher impedance response observed for NOMAF. In contrast, TFMAF shows the highest t_{Li+} value, suggesting more efficient Li^+ migration under polarization conditions. Together with its higher ionic conductivity, the elevated t_{Li+} of TFMAF helps explain its better rate capability and cycling stability compared with NOMAF among the flame-retardant electrolyte systems. Nevertheless, EDF still delivers superior overall electrochemical performance despite its lower ionic conductivity and t_{Li+} compared with TFMAF, indicating that bulk transport properties alone do not fully determine cell behavior. Interfacial charge-transfer kinetics, SEI/interphase composition, and electrolyte–electrode compatibility also play critical roles, as supported by the EIS/DRT and XPS analyses.

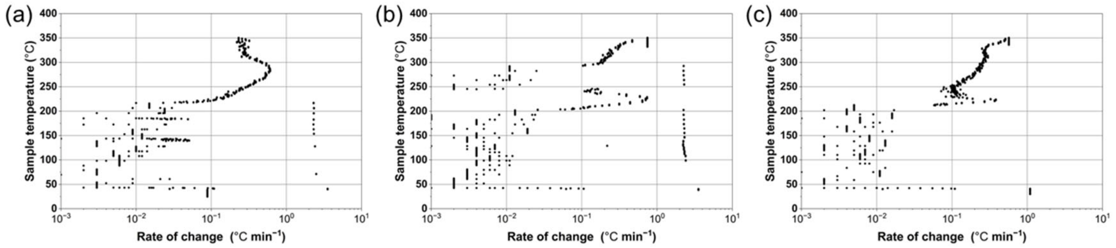

Figure S15. Accelerating rate calorimetry (ARC) profiles of (a) EDF, (b) NOMAF, and (c) TFMAF electrolytes, plotted as sample temperature (°C) versus the rate of temperature change (°C min^{-1}).

Table S1. Quantitative separation of irreversible loss and reversible capacity gain in different voltage windows.

Voltage window (V)	1st Discharge capacity (mAh g^{-1})	2nd Discharge capacity (mAh g^{-1})	Irreversible loss (mAh g^{-1})	Reversible capacity (mAh g^{-1})	Additional reversible gain vs 1.0–3.0 V (mAh g^{-1})
1.0–3.0	289–315	273–289	16–33	273–289	-
0.01–3.0	479–501	386–387	93–115	386–387	+97–113

The data clearly indicate that, although a portion of the initial capacity originates from irreversible processes—such as electrolyte reduction, solid electrolyte interphase (SEI) formation, and structural/electrochemical activation—a substantial fraction of the additional capacity remains reversible after the first cycle. Specifically, the stabilized reversible capacity in the second cycle (0.01–3.0 V) remains significantly higher (~386–387 mAh g^{-1}) than that obtained within the conventional voltage window (~273–289 mAh g^{-1}), corresponding to a net reversible capacity gain of 97–113 mAh g^{-1}. This additional reversible capacity accounts for approximately 25–29% of the theoretical capacity of $TiNb_2O_7$(TNO, ~387 mAh g^{-1}), demonstrating that the low-potential region provides a meaningful and sustainable enhancement in capacity, rather than being predominantly governed by irreversible side reactions.

Table S2. Second-cycle discharge capacities of EDF, NOMAF, and TFMAF measured at stepwise current densities (0.1, 0.5, 1, 3, 5, and 10 A g^{-1}) under two voltage windows (0.01–3.0 V and 1.0–3.0 V), along with the corresponding capacity retention values upon returning to 0.1 A g^{-1}.

Cut-off	Sample	2nd cycle discharge capacity at 0.1, 0.5, 1, 3, 5, 10 Ag^{-1} ($mAhg^{-1}$)	Capacity retention at 0.1Ag^{-1} (%)
0.01-3.0V	EDF	387, 283, 236, 166, 132, 81	80.3 (311 $mAhg^{-1}$)
	NOMAF	386, 258, 214, 144, 103, 53	77.4 (299 $mAhg^{-1}$)
	TFMAF	387, 270, 224, 154, 110, 57	77.5 (300 $mAhg^{-1}$)
1.0-3.0V	EDF	286, 241, 216, 163, 132, 67	91.6 (262 $mAhg^{-1}$)
	NOMAF	277, 231, 203, 119, 57, 13	98.1 (272 $mAhg^{-1}$)
	TFMAF	277, 226, 195, 116, 65, 14	90.6 (251 $mAhg^{-1}$)

Table S3. Discharge capacities measured at the 1st and 1,000th cycles and the corresponding capacity retention values of TNO nanofiber (NF) anodes in EDF, NOMAF, and TFMAF electrolytes at a high current density of 5 A g^{-1} under two voltage windows (0.01–3.0 V and 1.0–3.0 V vs. Li/Li^{+}). The data summarize long-term cycling performance and highlight the influence of electrolyte composition and operating voltage window on capacity retention behavior.

Cut-off	Sample	Discharge capacity at 5 Ag^{-1} ($mAhg^{-1}$)		Capacity retention (%)
		1st cycle	$1{,}000^{th}$ cycle	
0.01-3.0V	EDF	246	133	54
	NOMAF	202	67	33
	TFMAF	213	90	42
1.0-3.0V	EDF	214	97	45
	NOMAF	85	33	39
	TFMAF	76	34	45

Table S4. Room-temperature ionic conductivity, bulk resistance (R), separator thickness (d), and effective area (A) for EDF, NOMAF, and TFMAF electrolytes.

Electrolyte	R/ Ω	d/ cm	A/ cm^2	Ionic conductivity / mS cm^{-1}
EDF	3.394	0.017	2.01	2.49
NOMAF	6.639	0.017	2.01	1.27
TFMAF	2.895	0.017	2.01	2.92

References

1. Jo, H.; Kim, J.; Nguyen, D.-T.; Kang, K.K.; Jeon, D.-M.; Yang, A.-R.; Song, S.-W. Stabilizing the Solid Electrolyte Interphase Layer and Cycling Performance of Silicon–Graphite Battery Anode by Using a Binary Additive of Fluorinated Carbonates. *J. Phys. Chem. C* 2016, 120, 22466–22475.

2. Parimalam, B.S.; MacIntosh, A.D.; Kadam, R.; Lucht, B.L. Decomposition Reactions of Anode Solid Electrolyte Interphase (SEI) Components with $LiPF_6$. J. Phys. Chem. C 2017, 121, 22733–22738.
3. Wood, K.N.; Teeter, G. XPS on Li-Battery-Related Compounds: Analysis of Inorganic SEI Phases and a Methodology for Charge Correction. ACS Appl. Energy Mater. 2018, 1, 4493–4504.
4. Chen, Y.; Zhang, Y.; Niu, J.; Xu, H.; Dong, Z.; Xu, J.; Lei, C. Poly(Ether-Ester)-Based Solid Polymer Electrolytes with High Li-Ion Transference Number for High Voltage All-Solid-State Lithium Metal Batteries. *ACS Appl. Energy Mater.* 2023, 6, 3113–3125.
5. Li, A.; Xin, W.; Wang, Q.; Ai, W.; Han, W.; Yang, C.; Wang, Y.; Du, N.; Liu, C.; Zhang, Y.; Li, X.; Zhang, Y. Study on an Interpenetrating Artificial SEI for Lithium Metal Anode Modification and Fast Charging Characterization. *ACS Appl. Mater. Interfaces* 2024, 16, 65984–65992.
6. Yoo, D.-J.; Liu, Q.; Cohen, O.; Kim, M.; Persson, K.A.; Zhang, Z. Understanding the Role of SEI Layer in Low-Temperature Performance of Lithium-Ion Batteries. *ACS Appl. Mater. Interfaces* 2022, 14, 11910–11918.
7. S Sun, R.; Tao, Y.; Sun, H.; Chen, W.; Liu, G.; Yue, Y.; Hu, M.; Liu, M. Simple synthesis of $TiNb_6O_{17}$/C composite toward high-rate lithium storage. *J. Mater. Sci.* 2019, 54, 14825–14833.
8. Wu, Z.; Bak, S.-M.; Shadike, Z.; Yu, S.; Hu, E.; Xing, X.; Du, Y.; Yang, X.-Q.; Liu, H.; Liu, P. Understanding the Roles of the Electrode/Electrolyte Interface for Enabling Stable Li‖Sulfurized Polyacrylonitrile Batteries. *ACS Appl. Mater. Interfaces* 2021, 13, 31733–31740.
9. Zheng, X.; Wang, X.; Cai, X.; Xing, L.; Xu, M.; Liao, Y.; Li, X.; Li, W. Constructing a Protective Interface Film on Layered Lithium-Rich Cathode Using an Electrolyte Additive with Special Molecule Structure. *ACS Appl. Mater. Interfaces* 2016, 8, 30116–30125.
